# The Physiological MicroRNA Landscape in Nipple Aspirate Fluid: Differences and Similarities with Breast Tissue, Breast Milk, Plasma and Serum

**DOI:** 10.3390/ijms21228466

**Published:** 2020-11-11

**Authors:** Susana I. S. Patuleia, Carla H. van Gils, Angie M. Oneto Cao, Marije F. Bakker, Paul J. van Diest, Elsken van der Wall, Cathy B. Moelans

**Affiliations:** 1 Department of Pathology, University Medical Center Utrecht, Utrecht University, 3508 GA Utrecht, The Netherlands; s.i.schoutenpatuleia-7@umcutrecht.nl (S.I.S.P.); A.M.OnetoCao@umcutrecht.nl (A.M.O.C.); p.j.vandiest@umcutrecht.nl (P.J.v.D.); 2 Department of Medical Oncology, University Medical Center Utrecht, Utrecht University, 3508 GA Utrecht, The Netherlands; e.vanderwall@umcutrecht.nl; 3 Department of Epidemiology of the Julius Center for Health Sciences and Primary Care, University Medical Center Utrecht, Utrecht University, 3508 GA Utrecht, The Netherlands; c.vangils@umcutrecht.nl (C.H.v.G.); m.f.bakker-8@umcutrecht.nl (M.F.B.)

**Keywords:** miRNA, small RNA, biomarker, baseline, ranking, healthy, NGS, profiling, micro-array, NAF, serum, plasma, breast tissue, breast milk

## Abstract

Background: MicroRNAs (miRNAs) target 60% of human messenger RNAs and can be detected in tissues and biofluids without loss of stability during sample processing, making them highly appraised upcoming biomarkers for evaluation of disease. However, reporting of the abundantly expressed miRNAs in healthy samples is often surpassed. Here, we characterized for the first time the physiological miRNA landscape in a biofluid of the healthy breast: nipple aspirate fluid (NAF), and compared NAF miRNA expression patterns with publically available miRNA expression profiles of healthy breast tissue, breast milk, plasma and serum. Methods: MiRNA RT-qPCR profiling of NAF (*n* = 41) and serum (*n* = 23) samples from two healthy female cohorts was performed using the TaqMan OpenArray Human Advanced MicroRNA 754-Panel. MiRNA quantification data based on non-targeted or multi-targeted profiling techniques for breast tissue, breast milk, plasma and serum were retrieved from the literature by means of a systematic search. MiRNAs from each individual study were orderly ranked between 1 and 50, combined into an overall ranking per sample type and compared. Results: NAF expressed 11 unique miRNAs and shared 21/50 miRNAs with breast tissue. Seven miRNAs were shared between the five sample types. Overlap between sample types varied between 42% and 62%. Highly ranked NAF miRNAs have established roles in breast carcinogenesis. Conclusion: This is the first study to characterize and compare the unique physiological NAF-derived miRNA landscape with the physiological expression pattern in breast tissue, breast milk, plasma and serum. Breast-specific sources did not mutually overlap more than with systemic sources. Given their established role in carcinogenesis, NAF miRNA assessment could be a valuable tool in breast tumor diagnostics.

## 1. Introduction

MicroRNAs (miRNAs) are small, non-coding RNAs of ~22 nucleotides that regulate around 60% of messenger RNAs in humans [1]. MiRNAs are released into many biofluids as a result of cellular apoptosis or necrotic cell death, or by active secretion from their cells of origin, engulfed in extracellular vesicles such as exosomes or by forming complexes with RNA-binding proteins [2,3,4]. The fact that they are easily detectable in biofluids and remain stable during sample processing makes miRNAs suitable as upcoming disease biomarker candidates [4,5,6,7,8,9]. It has been established that miRNAs are involved in many signaling pathways and are associated with numerous disorders in the oncological, cardiovascular and neurological fields [10,11,12,13]. Yet, such findings are usually based on studies that solely focus on the differentially expressed miRNAs between two or more groups and fail to define the physiological miRNA landscape of the controls. It is, however, of utmost importance to establish and make available the baseline values of these biomarkers, especially given the potential clinical use of miRNAs. Such reference data can be established by investigating the most abundantly expressed miRNAs in healthy samples.

The establishment of such a baseline requires the use of large-scale profiling methodology for miRNA detection and quantification, such as RT-qPCR profiling, microarray and next-generation sequencing techniques [14]. These techniques facilitate simultaneous assessment of hundreds of miRNAs or even theoretically allow detection of all known 2656 mature miRNAs in *Homo sapiens* [15]. In establishing a baseline ranking of miRNAs, the sample source needs to be taken into account. Different body sources may have a different set of physiologically expressed miRNAs [16] and, in theory, liquid biopsies closest to the tissue of origin may best reflect its miRNA pattern. As such, we hypothesized that nipple aspirate fluid (NAF) is a potential useful source of miRNAs derived from the breast. NAF is secreted by the breast ducts of adult non-lactating women and is readily accessible by oxytocin-supported non-invasive vacuum aspiration, yielding sufficient material for molecular analysis [17,18,19,20,21]. NAF aspiration is associated with significantly less discomfort compared to mammography and MRI and may therefore represent a promising tool for breast cancer screening [20]. So far, characterization of the miRNAs present in NAF has never been reported. Such a characterization, together with a systematic comparison between healthy NAF and other breast specific and systemic healthy samples provides insight into the source specificity of miRNAs prior to breast cancer case-control studies.

This study focuses on defining the naturally occurring most abundantly expressed miRNAs in NAF compared to other (non-)liquid sample types, namely breast tissue, breast milk, plasma and serum, as measured by multi-targeted profiling techniques in our cohorts and in other studies. Data were in the majority extracted from the literature and combined with data from our own discovery experiments with NAF and serum. Breast tissue was included given that this sample has a well-established diagnostic role in breast disease and is a specific source of breast-derived biomarkers. NAF and breast milk were included as these are likely liquid representatives of the breast microenvironment. The systemic readily available biofluids plasma and serum, which are the most commonly used liquid biopsies in research and the clinic, allowed comparison with site-specific biosamples. 

## 2. Results

### 2.1. Study Selection for Ranking of Breast Tissue, Breast Milk, Plasma and Serum

**Figure 1:**
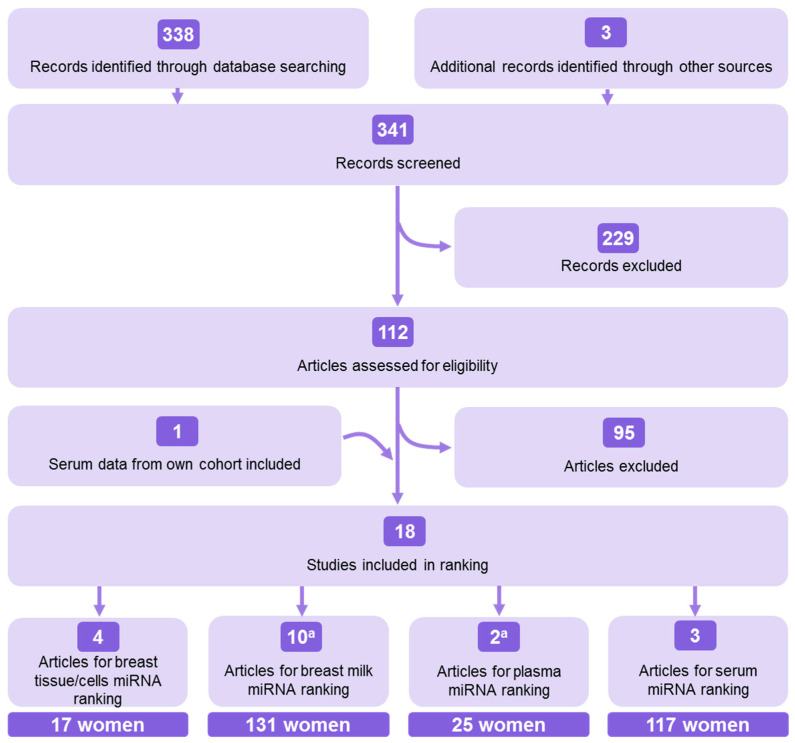
Flowchart of the study selection strategy used for miRNA ranking in four biosamples: breast tissue, breast milk, plasma and serum. Adapted from Preferred Reporting Items for Systematic Reviews and Meta-Analyses (PRISMA) guidelines [39]. Detailed flowcharts are shown in Supplementary Methods. The total number of women included in the selected studies per sample type was added underneath. a. One article provided data for the breast milk miRNA ranking and for the plasma miRNA ranking.

In total, 341 records were screened, out of which 17 studies were included for miRNA ranking per biosample (four for breast tissue/cells [22,23,24,25], ten for breast milk [26,27,28,29,30,31,32,33,34,35], two for plasma [27,36] and two for serum [37,38]). One article reported data for breast milk as well as plasma ranking [27]. Additionally, data from our own serum cohort were included in the ranking. This led to a total of 18 included studies for the ranking of these four biosamples. The flow of the search strategy is shown in Figure 1 and presented in detail in Supplementary Methods.

MiRNA expression data were derived from a total of 331 women. In the included literature studies, women were on average around 25 years younger in the breast milk and plasma studies (mean 30.3 and 32.93, respectively, based on available data from merely 28/173 women) compared to the women in the serum and NAF studies (mean 58.69 and 55, respectively, based on data from 158/158 women). The limited anthropomorphic characteristics of the healthy women that could be extracted from the included studies are shown in Supplementary Table S3. Most of the studies applied RNA sequencing techniques, only one study used a microarray platform [23] and two studies and our cohort were based on miRNA profiling with a wide panel of miRNAs (667–754 miRNA targets) [35,36]. A wide variety of (mi)RNA isolation kits were used. Detailed technical information is shown in Supplementary Table S3. 

### 2.2. MiRNA Expression Profiling in Nipple Aspirate Fluid and Serum and Ranking

The concentration of total RNA extracted from NAF varied between 3 and 134 ng/µL, with a mean of 42.2 ng/µL (SD 35.2). Nanoliter fluidics-based miRNA profiling was successful in all samples, with 296 out of the 754 miRNAs in the profiling panel (39%) determined with mean 10 ≤ CRT ≤ 29. To add perspective, 29/754 (4%) miRNAs were detected in all samples (*n* = 41), 85/754 (11%) miRNAs were detected in 90% of the samples (*n* ≥ 37) and 186/754 miRNAs (25%) were detectable in 50% of the samples (*n* ≥ 20). Mean and median expression for each miRNA present in at least 10/41 NAF samples is available in Supplementary Table S1. NAF ranking was performed with miRNAs detected in at least 80% of the samples (*n* ≥ 33), which was a total of 118/754 miRNAs (16%). The 50 most highly expressed microRNAs discovered in NAF are presented in Table 1. Correlation between profiling and individual qPCR technical validation for three miRNAs in the top 50 was highly significant (*p* < 0.0001; R squared between 0.6344 and 0.8644; Supplementary Figure S1). 

In serum samples, total RNA concentration varied between 52 and 73 ng/µL, with a mean of 61.9 ng/µL (SD 6.1). Using the same 754-miRNA profiling panel as for the NAF samples, 89/754 (12%) miRNAs were detected in at least 90% of the samples (*n* ≥ 20), 110/754 miRNAs (15%) were detected in 80% of the samples (*n* ≥ 18) and 153/754 miRNAs (20%) were detected in 50% of the samples (*n* ≥ 11). Similarly to the NAF samples, an 80% detection cut-off was applied to select the miRNA data used for serum ranking.

### 2.3. Roughly Half of the Highly Expressed miRNAs in Breast Tissue and Nipple Aspirate Fluid Are also Present in Other Samples

The lists of the 50 most abundant miRNAs per healthy sample type (breast tissue, NAF, breast milk, plasma and serum) are shown in Table 1 and the rankings per individual study are available in Supplementary Table S1. A total of 110 unique miRNAs were found when comparing the top 50 across all overall rankings. Out of these, only seven miRNAs were highly ranked in all five sample types, namely hsa-miR-148a-3p, hsa-let-7b-5p, hsa-miR-26a-5p, hsa-miR-24-3p, hsa-let-7g-5p, hsa-miR-21-5p and hsa-miR-22-3p.

**Figure 2:**
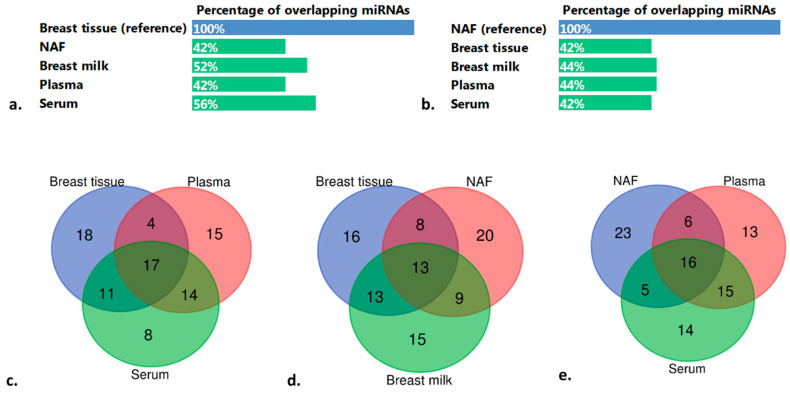
Overlapping miRNAs between sample types. (**a**,**b**): percentage of overlapping miRNAs between the top 50 of breast tissue (reference, **a**) and nipple aspirate fluid (NAF; reference, **b**) and the top 50 of the other four sample types. (**c**,**d**): number of overlapping miRNAs between the top 50 of breast tissue and the top 50 of two systemic biofluids (plasma and serum, **c**) and two breast-derived biofluids (NAF and breast milk, **d**). (**e**): number of overlapping miRNAs between de top 50 of NAF and the top 50 of two systemic biofluids: plasma and serum. The list of shared miRNAs is shown in Supplementary Table S4 and Supplementary Figure S2. A 5-way Venn diagram is shown in Supplementary Figure S4.

The number of overlapping miRNAs between breast tissue and each biofluid was investigated. With the top 50 of breast tissue as a reference, the highest number of overlapping miRNAs was found in serum (*n* = 28; 56%), followed by breast milk (*n* = 26) and subsequently NAF (*n* = 21) and plasma (*n* = 21) (Figure 2a). A total of 17 miRNAs was found overlapping between breast tissue and the two systemic biofluids serum and plasma (Figure 2c). A somewhat lower number of miRNAs (*n* = 13) overlapped between breast tissue and the two breast-derived biofluids (NAF and breast milk) (Figure 2d). 

When the top 50 of NAF was used as a reference compared to each of the other sample types, a comparable number of overlapping miRNAs was found, namely 21–22 miRNAs (42–44%) (Figure 2b). Comparing the top 50 of NAF and both systemic biofluids, a total of 16 miRNAs overlapped (Figure 2e). Five miRNAs were exclusively shared between NAF and breast tissue, namely hsa-miR-221-3p, hsa-miR-205-5p, hsa-miR-125b-5p, hsa-miR-145-5p and hsa-miR-130a-3p (Supplementary Table S4). Four miRNAs were exclusively shared between NAF and breast milk, namely hsa-miR-181b-5p hsa-miR-193b-3p hsa-miR-200c-3p and hsa-miR-200b-3p. In Supplementary Table S4 and Supplementary Figure S2, these and additional overlapping comparisons are presented. In general, the overlap between the top 50 ranking of individual sample types varied marginally between 42–62%. The greatest overlap was observed between serum and plasma (62%). The overlap amongst breast-specific sample types varied between 42–52% whereas the overlap between breast-specific samples and systemic sample types was 42–56%. 

Non-overlapping miRNAs were also observed. A list of these potentially unique biosample-enriched miRNAs is presented in Table 2. NAF and breast tissue contained the highest number of unique miRNAs (*n* = 11 and *n* = 9, respectively), while serum had the lowest number of unique miRNAs (*n* = 5).

### 2.4. The Majority of Abundant miRNAs in Nipple Aspirate Fluid Are Immune-Related and Established Tumor Suppressors

To obtain insight into the biological processes in which the most highly expressed NAF miRNAs are involved, miRPathDB v2.0 [40,41] was used. Kyoto Encyclopedia of Genes and Genomes (KEGG) pathway analysis revealed that the top 20 of the most highly expressed miRNAs in NAF are annotated for, amongst others, “MicroRNAs in cancer”, “Pathways in cancer”, “Focal adhesion” and “PI3K/AKT signaling pathway”. Interestingly, WikiPathway showed that most of the miRNAs in NAF have been categorized as being involved in the “breast cancer pathway”. Moreover, these miRNAs were annotated for “regulation of cell population proliferation”, “response to organic substance”, “cell death” and “(cellular) protein metabolic process” according to Gene Ontology (GO) biological processes enrichment analysis. The three pathway analyses are shown in Supplementary Figure S3. In Supplementary Table S7, the unique physiological roles and pathway involvement to each set of specimen-specific miRNAs (Table 2) and for the seven shared miRNAs (Table 1, bold) are shown. Interestingly, “integrated breast cancer pathways” are related to the majority of unique NAF-miRNAs and “breast cancer pathways” are related to unique breast milk miRNAs and unique NAF-miRNAs.

Additionally, of the 50 most abundant miRNAs in NAF, 39 (78%) have been annotated as immune-related miRNAs, i.e., related to immunity pathways and/or targets reported to be involved in immunity (Supplementary Table S5). All of the 10 most highly expressed miRNAs in NAF have been listed as immune-related miRNAs.

Lastly, a target analysis was performed for the top 20 NAF miRNAs. Common established targets were BCL2 (a suppressor of apoptosis, targeted by 9/20 miRNAs), PTEN (a tumor suppressor that antagonizes the PI3K-AKT signaling pathway, targeted by 8/20 miRNAs), VEGFA (a proto-oncogene involved in angiogenesis, targeted by 8/20 miRNAs), BMI1 (a proto-oncogene involved in stem cell renewal and DNA repair, targeted by 7/20 miRNAs), CCND1 (an oncogene involved in cell cycle progression, targeted by 7/20 miRNAs), TP53 (a tumor suppressor that induces growth arrest and induces apoptosis, targeted by 6/20 miRNAs) and SP1 (a proto-oncogenic transcription factor, targeted by 5/20 miRNAs), amongst others (IGF1R, KRAS, ZEB2, AKT2, CDK6, MYC and RECK). Depending on their targets, miRNAs can be classified into tumor suppressor miRNAs or oncogenic miRNAs. Notably, 15 (75%) of the 20 most highly expressed miRNAs in NAF have an established tumor suppressor role in breast cancer. Supplementary Table S6 provides a summary of the most relevant established targets and cellular processes in which each of the top 20 NAF miRNAs has been shown to be involved in.

## 3. Discussion

MiRNAs have the potential to serve as novel clinical biomarkers as they can reflect subtle changes occurring in easily accessed biofluids, better known as liquid biopsies. Thousands of research papers related to miRNAs have been published in relation to disease, usually focusing on the significance and fold change of differentially expressed miRNAs between cases and controls, sometimes correlating with endpoints such as overall survival and prognosis [42,43,44]. However, reporting the baseline miRNA levels in healthy controls is often surpassed and not systematically reported [45]. As a consequence, data on normally expected miRNAs in the healthy state are very limited. Here, using literature data supplemented with our own data, we present the 50 most highly expressed miRNAs in five healthy sample types, namely breast tissue, and the liquid biosamples NAF, breast milk, plasma and serum. Furthermore, this is the first study defining the physiological miRNA landscape in NAF and its overlap with other biosample types.

To establish a baseline ranking of highly expressed miRNAs, a systematic literature search was applied to ensure that all the relevant articles were found. As quantitative measurements of miRNAs across studies could not be compared due to the application of different techniques and platforms, a ranking from 1 to 50 was established. The overall overlap analysis of the five top 50 miRNA rankings showed that merely seven out of the 110 unique miRNAs were shared between the interrogated sample types, confirming the importance of biosample or biofluid choice for biomarker analysis. These seven shared microRNAs across all sample types overlapped for a great number of unique pathways (Supplementary Table S7), which could imply that these microRNAs are abundant in the whole body and involved in many, diverse and, hence, aspecific pathways. In addition, the overlap analyses unexpectedly showed that the highly expressed miRNAs in breast-specific sources did not mutually overlap more than with systemic sources. For instance, the greatest miRNA overlap between breast tissue and another sample type was seen with serum (28 miRNAs). This either suggests that most miRNAs derived from breast tissue are released into this blood fraction, or that other non-investigated tissues might express overlapping miRNAs that are released into the bloodstream. An additional comparison with a miRNA ranking from minimally one other healthy tissue would provide insight into the latter. A publicly available “healthy genome atlas” for tissue and liquid biopsy data would facilitate such miRNA specificity analyses.

Given that this is the first study presenting data on miRNA profiling in healthy NAF, detailed analyses from the perspective of NAF were performed. Here we show that miRNA profiling using a wide panel of 754 miRNA targets was successful in all NAF samples. Moreover, the technical validation of three miRNAs demonstrated reproducibility. An overlap analysis using the highly expressed miRNAs in NAF as a reference in comparison to the other samples showed that a minority of NAF miRNAs are also abundant in the four other sample types (21–22/50 miRNAs), irrespective of it being a liquid or solid sample, a local or a peripheral/systemic sample. 

Exploration of the relevance of the most highly expressed miRNAs in NAF revealed that the majority of NAF miRNAs are tumor suppressors involved in breast carcinogenesis pathways. The highest ranked miRNA in NAF was hsa-miRNA-205-5p, also positioned in the top 50 of breast tissue. This tumor suppressor miRNA, known to be exclusively expressed in myoepithelial cells, has been associated with the regulation of adherens junctions and focal adhesion. As such, downregulation of hsa-miRNA-205-5p leads to tumor invasion [46,47,48]. Two shared miRNAs between NAF and milk were hsa-miRNA-200b-3p and hsa-miRNA-200c-3p, belonging to the tumor suppressor miRNA-200 family [49,50]. Hsa-miRNAs 200b/c-3p directly target E-cadherin transcriptional repressors ZEB1/ZEB2, thereby inhibiting epithelial–mesenchymal transition (EMT) [51,52,53,54,55]. Downregulation of these miRNAs has been associated with breast cancer metastasis [56].

A total of 11 NAF miRNAs were not expressed in the top 50 of the other sample types and are therefore likely NAF-enriched. The most highly ranked amongst them, hsa-miR-203a-3p (ranked second) is an established tumor suppressor that has been shown to compromise cellular migratory and invasive capacity in vitro, and tumor initiation and metastasis in vivo [57]. Downregulation of hsa-miR-203 has been associated with increased extracellular matrix stiffness [58]. Increased breast stiffness is visible in radiological findings (“dense breasts”), and is a known relevant risk factor for breast cancer development, biologically still far from understood [59,60]. Other highly ranked miRNAs unique for the top 50 of NAF were hsa-miR-15a-5p and hsa-miR-193b-3p. Likewise, their downregulation has been related to breast cancer by influencing migration [61], cell cycle progression and apoptosis [62,63]. Not only these miRNAs, but the majority of highly ranked miRNAs in NAF are involved in cellular proliferation, differentiation, apoptosis and/or target commonly known mRNAs involved in carcinogenesis, like PTEN, BCL2 and VEGFA. Moreover, all the top 10 miRNAs in NAF play a prominent role in immunity, which is of increasing relevance in the context of breast cancer immunotherapy treatment [64,65,66]. Altogether, this shows that miRNAs detected in NAF have established roles in breast carcinogenesis. Given their source specificity and tumor suppressor role, they might be overlooked or detected with lower sensitivity in case-control studies that use other sample sources.

From the above, it can be concluded that having insight into the miRNA patterns of normal tissues and biological fluids is key to be able to correctly interpret miRNA patterns as biomarkers of disease. Nonetheless, this study has some limitations. First, with a range of 1 to 10 studies per sample type for the overall rankings, a relative low number of studies was included. This was the result of strict inclusion and exclusion criteria for article selection. For instance, it was chosen to select studies with miRNA data for women, as female gender was the only possible miRNA confounding variable [67] that could be filtered a priori. MiRNA data from The Cancer Genome Atlas (TCGA [68,69]) was queried but not used because miRNA data from normal breast tissue adjacent to tumor is believed to have a different profile compared to normal healthy breast tissue [70]. Moreover, many of the studies were excluded because the individual quantitative data per miRNA in the healthy cohort was not provided (62 studies out of the 92 excluded based on full text, Supplementary Methods), which strengthens the reason and need for the present study. Still, the data for the rankings are derived from an ample number of women, namely 331 women.

Second, the presented ranking positions per miRNA may not be entirely accurate as these positions are dependent on the ranking strategy, isolation method [71], sample pooling, anthropomorphic characteristics of the cohort and/or cut-off values for miRNA selection. To mention a few possible confounding factors, for the overall breast milk ranking in this study, a combination of the individual rankings per milk fraction (lipid fraction, skim milk, cells or exosomes) was applied, and for the ranking of breast tissue, two in vitro studies using mammary epithelial cells were integrated in the ranking. Regarding anthropomorphic characteristics, based on available data from a minority of women, the breast milk and plasma cohorts in this study were collected from younger women compared to the cohorts for the other samples (Supplementary Table S3), which may have altered miRNA expression [67,72]. Another baseline characteristic relevant for women in the context of breast cancer is mammographic density, which was only known within our own NAF cohort. NAF samples were derived from women with either extremely high or very low MD, together with a 20% prevalence in the female population [73]. Whether this influences the presented data is unclear as the influence of breast density on miRNA expression has not been studied yet. Regarding cut-off values, in the NAF and serum cohorts that produced our own data, miRNAs had to be expressed in at least 80% of the interrogated samples to qualify for ranking selection; this was probably not applied by others, or at least not reported. Adaptation of any of these variables may shuffle individual miRNAs to another ranking position. A similar study comparing these (and more) sample types from the same subjects would be ideal, but challenging with the unique combination of samples chosen for this study. 

In conclusion, this is the first study to establish and compare the physiological miRNA expression pattern in three breast-specific and two systemic healthy sample types. A publicly available genomic data atlas repository for healthy samples currently lacks and should be created to allow further comparisons and evaluations, such as the investigation of sample type (a)specificity of potential biomarker miRNAs. Such data needs to be accompanied with a clear definition of what is healthy, with anthropomorphic characteristics, sample fraction specification and should be based on uniform sample processing, techniques and platforms. Most importantly, this is the first study that characterizes the unique physiological NAF-derived miRNA landscape. Surprisingly, in terms of abundant physiological miRNAs, healthy NAF does not seem a better representative of the breast microenvironment than commonly used biofluids such as serum or plasma. Nevertheless, healthy NAF contains measurable amounts of miRNAs with established roles in breast carcinogenesis, some of them likely enriched compared to serum and plasma. As such, there is a possible clinical applicability of NAF-derived miRNAs that should further be explored.

## 4. Materials and Methods 

### 4.1. Nipple Aspirate Fluid and Serum Collection and Processing

NAF had already been collected from 41 women in the context of the Dutch nationwide multicenter Dense tissue and Early breast Neoplasm ScrEening (DENSE) trial (NCT01315015 [74,75]). Samples were derived from women with extremely high (D, *n* = 21) or very low (A, *n* = 20) mammographic density (MD), which are the two extreme categories within the four-category MD spectrum (A—D [76]). Serum was obtained from 23 women included in the healthy cohort of another NAF study (NL41845.041.12 [77]). Hereafter, these cohorts will be referred to as the “NAF cohort” and the “serum cohort”, respectively. These studies were approved by the Institutional Review Boards of the UMC Utrecht and other participating hospitals (study number 12-495 approved on January 29 2013) and the UMC Utrecht Biobank Research Ethics Committee (TCBio; biobank study number 14-467 approved on 17 June 2015). Informed consent was obtained from all participating women. 

NAF samples were collected between June 2015 and March 2016, and serum samples were collected between August 2017 and November 2019. At the time of sample collection, all participants were healthy and had a minimum age of 50 and 45 years old according to inclusion criteria for the NAF and serum cohorts, respectively. For the NAF cohort, healthy was defined as not having breast abnormalities on recent mammography and/or magnetic resonance imaging. For the serum cohort, this was defined as not having breast cancer and not having an increased risk for developing breast cancer according to personal and familial history. The NAF samples were acquired after nasal oxytocin administration as described previously [19,20]. Bilaterally acquired samples were conserved in a buffer solution (50 mM Tris pH 8.0, 150 mM NaCl, 2mM EDTA). Serum samples were acquired by phlebotomy in the median cubital vein. After collection, serum was processed by centrifugation at 300× *g* for 15 min. Aliquots were immediately stored at −80 °C until use.

### 4.2. Extraction and Quantification of RNA from Nipple Aspirate Fluid and Serum

Total RNA extraction from pooled NAF samples (intra-individual samples from left and right breast combined) and serum was performed according to the manufacturer’s protocol using the AllPrep DNA/RNA/miRNA Universal Kit (Qiagen, Hilden, Germany) and the miRNeasy serum/plasma kit (Qiagen), respectively. All extractions were performed starting with 20 µL of pooled NAF and 200 µL of serum. For the NAF samples, non-human ath-mir-159 (with a 5′ phosphate) was spiked in as quality control at 300 pg by pre-mixing with RLT plus lysis buffer. For the serum samples, 50 pg non-human ath-mir-159 (with a 5′ phosphate) and 1 μg MS2 carrier RNA (Roche) were spiked in as quality control after Qiazol incubation. RNA was eluted in 30 µL or 17 µL of RNAse-free water for the NAF and serum samples, respectively. The concentration of the extracted RNA was measured by Qubit 3.0 (ThermoFisher Scientific, MA, USA) fluorometric quantification. All RNA samples were then stored at −80 °C until further analysis.

### 4.3. Reverse Transcription, Preamplification and Taqman OpenArray Profiling Analysis of Nipple Aspirate Fluid and Serum

The expression levels of 754 human mature miRNAs (Supplementary Methods) that have been functionally validated with miRNA artificial templates were profiled using the fixed-content TaqMan OpenArray Human Advanced MicroRNA Panel on a QuantStudio 12 K Flex system (ThermoFisher Scientific, MA, USA). According to the manufacturer’s instructions, total RNA (8 ng from NAF and 2 µL from serum) was first poly-A tailed, and after adaptor ligation and reverse transcription, pre-amplified for 19 cycles. The pre-amplification product was subsequently diluted 20× in 0.1× TE buffer pH 8.0. The samples were loaded from a 384-well plate onto the TaqMan OpenArray Human Advanced MicroRNA Panel array slide using the OpenArray AccuFill system. Relative threshold values (CRT [78]), proven to be more robust than baseline threshold values for analyzing data generated using nanoliter fluidics-based OpenArray plates, were automatically calculated using the ThermoFisher Cloud system [79]. Analysis settings included the following restrictions: a minimum CRT of 10, a minimum AMPSCORE (low signal in linear phase) of 1, a minimum calculated confidence in the quantification cycle (CQCONF) (Cq) value of 0.6, a maximum CRT of 28 with inclusion of maximum CRT in calculations. Additionally, all miRNA amplification plots were visually inspected on curve shape and signal timing.

A technical validation (i.e., quality control of NAF profiling data using the same samples) was performed using individual Taqman advanced miRNA assays hsa-miR-29a-3p, hsa-miR-324-5p and hsa-miR-181a-5p according to the manufacturer’s instructions. Pearson correlation was calculated and graphically visualized using GraphPad 8.0 for Windows (San Diego, CA, USA).

### 4.4. Literature Search: Search Strategy, Study Selection and Data Extraction

Four systematic searches were performed on PubMed for studies published up until May 2020 for samples of normal breast tissue or cells, breast milk, plasma and serum. Each search comprised words and synonyms of the biosample and of “microRNA”, “profiling techniques” and “healthy women”. Only those papers in which extensive miRNA profiling was performed either by deep sequencing, microarray or RT-qPCR profiling on either of these samples were selected for further ranking and comparison. Full syntaxes and detailed criteria for inclusion and exclusion are presented in Supplementary Methods. Two reviewers (S.I.S.P. and A.M.O.C.) independently screened the titles, abstracts and full texts of the studies retrieved from the searches. Any disagreement was solved through discussion to reach a consensus. In total, 17 studies and our own serum and NAF data were selected to establish rankings of the most highly expressed miRNAs per biosample. The process of article selection followed adapted Preferred Reporting Items for Systematic Reviews and Meta-Analyses (PRISMA) guidelines [39] and is presented in Figure 1 and detailed in Supplementary Methods. Quality assessment is one of the steps of the PRISMA guidelines, but was not performed as the studies were not diagnostic, therapeutic nor prognostic and hence did not fit to any of the existing quality assessment tools [80]. Extracted data of the selected articles included normalized read counts, units of fluorescence intensity, and C(R)T values of individual miRNAs measured in the healthy population, anthropomorphic characteristics of the healthy cohort (such as age, body mass index and breast density), details concerning sample fraction and sample processing, and techniques of choice used for (mi)RNA isolation, miRNA detection and quantification.

### 4.5. MiRNA Ranking 

Extracted data of the selected articles included normalized read counts (reads per kilobase million, transcripts per kilobase million, counts per million and others), units of fluorescence intensity, relative abundance and C(R)T values of individual miRNAs measured in the healthy population. This heterogeneity of measures used across studies, including studies within one sample type, led us to apply the strategy of developing a top 50 rank. Given that 2656 microRNAs are known [15], highlighting the top 50 miRNAs was considered reasonable to highlight the most abundant miRNAs in each sample type. 

To perform a miRNA ranking of the top 50 most highly expressed miRNAs in NAF samples, miRNAs detected in ≥80% of the NAF samples were selected. Secondly, miRNAs were orderly ranked based on their mean CRT values. The same strategy was applied to the miRNA profiling expression data from serum samples. This serum ranking was then combined with the serum rankings acquired from the literature into an overall serum ranking.

MiRNA data was retrieved from selected articles to perform a ranking of the top 50 most abundantly expressed miRNAs in breast tissue, breast milk, plasma and serum. As a first step, the miRNAs were ranked from highest to lowest expression (Supplementary Table S1). Listed miRNAs that were retracted from miRBase due to misclassification were excluded from the rankings, if present. Then, the rankings of the individual studies were combined into overall rankings per biosample. The formulas applied for the overall ranking weighted in the number of samples used in each study and the top 50 rank number (the consistency) across studies per miRNA (Supplementary Table S2). This ranking formula is similar to the ranking method applied for meta-analysis of miRNA case-control studies developed by Griffith et al. [81] and Chan et al. [82], and used by many for miRNA profiling studies [83,84,85,86,87], which focuses on consistency in direction of expression change, frequency of reporting a particular miRNA, total sample size for consistently reported miRNAs and average fold change. Afterwards, miRNAs in the overall rankings for breast tissue, breast milk, plasma and serum that were not present in the 754-panel used for the NAF profiling from own data were excluded for comparative analysis. This was solely the case for one miRNA in the plasma ranking (hsa-miR-135a-3p). Consequently, the plasma top 50 was supplemented with the 51^st^ most highly expressed miRNA, allowing a fair ranking comparison.

### 4.6. Ranking Comparison

MiRNA nomenclature was adapted for consistency across sample type rankings into the most recent nomenclature according to miRBase (v22) [88,89]. The number of overlapping miRNAs across sample types was then displayed in Venn diagrams using the web tool VENN [90]. GraphPad Prism 8.0 for Windows (San Diego, CA, USA) was used for graphical visualization of the ranking ranges of the top 20 miRNAs in breast tissue in the top 50 of the other four biosamples.

### 4.7. Physiological Roles, Pathway and Target Analysis of miRNAs Detected in Nipple Aspirate Fluid

Physiological roles and pathway involvement of NAF-derived miRNAs were retrieved from miRPathDB v2.0 [91] using the KEGG, WikiPathway and GO databases. Stringent criteria for miRPathDB query included selection of data based on strong experimental evidence and with at least 10 significant miRNAs per pathway. Involvement of these miRNAs in the immune processes was cross-checked with the Pathway Central database (SABiosciences, MD, USA) and Abcam’s Multiplex Circulating miRNA Immunology Fixed Panel (ab204064). Experimentally validated common targets of the NAF top 20 miRNAs were obtained from miRTarBase v7 [92]. Again, strict criteria were applied, namely that common mRNAs needed to be targeted by at least five miRNAs based on strong experimental evidence, which was defined as data derived from luciferase reporter assays or Western blot experiments.

### 4.8. Data Sharing Statement

The authors confirm that the data supporting the findings of this study are available within the article and its supplementary materials.

## Figures and Tables

**Table 1:** Ranking of the 50 most highly expressed miRNAs in five healthy sample types. Individual rankings per study are presented in Supplementary Table S1. MiRNAs in bold are abundant in all interrogated sample types. In Supplementary Table S7, the unique physiological roles and pathway involvement for the 7 shared miRNAs (bold) are shown.

Ranking	Breast Tissue [22,23,24,25]	Nipple Aspirate Fluid (This Study)	Breast Milk [26,27,28,29,30,31,32,33,34,35]	Plasma [27,36]	Serum ([37,38] and This Study)
1	**hsa-miR-24-3p**	hsa-miR-205-5p	**hsa-miR-148a-3p**	hsa-miR-451a	hsa-miR-451a
2	**hsa-miR-21-5p**	hsa-miR-203a-3p	**hsa-miR-22-3p**	hsa-miR-92a-3p	hsa-miR-92a-3p
3	hsa-miR-23a-3p	hsa-miR-26b-5p	hsa-miR-146b-5p	**hsa-miR-21-5p**	**hsa-miR-26a-5p**
4	hsa-let-7f-5p	hsa-miR-221-3p	hsa-miR-30d-5p	hsa-miR-126-3p	**hsa-miR-22-3p**
5	**hsa-let-7b-5p**	**hsa-let-7b-5p**	hsa-miR-30a-5p	hsa-miR-320a-3p	hsa-miR-25-3p
6	**hsa-miR-22-3p**	hsa-miR-27a-3p	**hsa-miR-26a-5p**	hsa-miR-122-5p	hsa-miR-423-5p
7	hsa-let-7a-5p	hsa-miR-451a	hsa-miR-375-3p	**hsa-let-7b-5p**	**hsa-miR-21-5p**
8	hsa-miR-30a-5p	hsa-miR-92a-3p	hsa-let-7a-5p	hsa-miR-191-5p	hsa-miR-16-5p
9	hsa-miR-125b-5p	hsa-miR-16-5p	hsa-miR-200a-3p	hsa-miR-223-3p	**hsa-miR-148a-3p**
10	**hsa-let-7g-5p**	**hsa-let-7g-5p**	hsa-miR-141-3p	**hsa-miR-24-3p**	hsa-let-7a-5p
11	hsa-miR-143-3p	**hsa-miR-24-3p**	**hsa-miR-21-5p**	hsa-miR-16-5p	hsa-let-7f-5p
12	hsa-miR-451a	hsa-miR-200c-3p	hsa-let-7f-5p	hsa-miR-19b-3p	hsa-miR-27a-3p
13	hsa-miR-99a-5p	hsa-miR-200b-3p	hsa-miR-181a-5p	hsa-miR-486-5p	hsa-miR-122-5p
14	hsa-miR-320a-3p	hsa-miR-99a-5p	hsa-miR-30b-5p	hsa-miR-150-5p	**hsa-let-7b-5p**
15	hsa-miR-145-5p	**hsa-miR-21-5p**	**hsa-let-7b-5p**	hsa-miR-17-5p	hsa-miR-142-5p
16	hsa-miR-205-5p	hsa-miR-92b-3p	**hsa-let-7g-5p**	hsa-miR-484	hsa-miR-30d-5p
17	hsa-miR-126-3p	hsa-miR-15a-5p	hsa-miR-92a-3p	hsa-miR-106a-5p	hsa-miR-181a-5p
18	**hsa-miR-26a-5p**	hsa-miR-193b-3p	hsa-miR-191-5p	hsa-miR-20a-5p	hsa-miR-126-3p
19	hsa-miR-19b-3p	hsa-miR-30b-5p	hsa-miR-320a-3p	hsa-miR-146a-5p	hsa-miR-191-5p
20	hsa-let-7i-5p	hsa-miR-29a-3p	hsa-miR-182-5p	**hsa-miR-26a-5p**	hsa-let-7i-5p
21	hsa-let-7c-5p	hsa-miR-34a-5p	hsa-miR-423-5p	hsa-miR-25-3p	hsa-miR-101-3p
22	**hsa-miR-148a-3p**	**hsa-miR-26a-5p**	hsa-miR-99b-5p	hsa-miR-222-3p	**hsa-miR-24-3p**
23	hsa-miR-199a-3p	hsa-miR-125b-5p	hsa-miR-200c-3p	hsa-miR-342-3p	**hsa-let-7g-5p**
24	hsa-miR-101-3p	**hsa-miR-22-3p**	hsa-let-7i-5p	hsa-miR-30a-5p	hsa-miR-26b-5p
25	hsa-miR-100-5p	hsa-miR-365a-3p	hsa-miR-200b-3p	hsa-miR-126-5p	hsa-miR-146a-5p
26	hsa-miR-29a-3p	**hsa-miR-148a-3p**	hsa-miR-101-3p	hsa-miR-30c-5p	hsa-miR-486-5p
27	hsa-miR-103a-3p	hsa-miR-17-5p	**hsa-miR-24-3p**	hsa-miR-142-3p	hsa-miR-223-3p
28	hsa-let-7d-5p	hsa-miR-20a-5p	hsa-miR-27b-3p	hsa-miR-30d-5p	hsa-miR-144-3p
29	hsa-miR-125a-5p	hsa-miR-652-3p	hsa-miR-29a-3p	hsa-miR-30e-5p	hsa-miR-143-3p
30	hsa-miR-141-3p	hsa-miR-144-3p	hsa-miR-99a-5p	hsa-miR-30b-5p	hsa-miR-30e-5p
31	hsa-miR-199b-3p	hsa-miR-125a-5p	hsa-miR-429	hsa-miR-192-5p	hsa-miR-320a-3p
32	hsa-miR-199a-5p	hsa-miR-210-3p	hsa-miR-16-5p	hsa-miR-425-5p	hsa-miR-103a-3p
33	hsa-miR-181a-5p	hsa-miR-25-3p	hsa-miR-378a-3p	hsa-miR-423-5p	hsa-miR-10b-5p
34	hsa-miR-30b-5p	hsa-miR-361-5p	hsa-miR-103a-3p	hsa-let-7e-5p	hsa-miR-423-3p
35	hsa-miR-497-5p	hsa-miR-29b-3p	hsa-miR-186-5p	hsa-miR-106b-5p	hsa-miR-126-5p
36	hsa-miR-30e-5p	hsa-miR-195-5p	hsa-miR-151a-3p	**hsa-miR-148a-3p**	hsa-miR-27b-3p
37	hsa-miR-140-3p	hsa-miR-181b-5p	hsa-miR-10a-5p	hsa-miR-19a-3p	hsa-miR-16-2-3p
38	hsa-miR-27a-3p	hsa-miR-30c-5p	hsa-miR-25-3p	hsa-let-7i-5p	hsa-miR-150-5p
39	hsa-miR-130a-3p	hsa-miR-342-3p	hsa-miR-30e-5p	hsa-miR-378a-3p	hsa-miR-92b-3p
40	hsa-miR-191-5p	hsa-miR-181a-5p	hsa-miR-181b-5p	**hsa-miR-22-3p**	hsa-miR-484
41	hsa-miR-195-5p	hsa-miR-223-3p	hsa-miR-125a-5p	hsa-miR-146b-5p	hsa-let-7d-5p
42	hsa-miR-939-5p	hsa-miR-146a-5p	hsa-miR-30c-5p	hsa-miR-151a-3p	hsa-miR-151a-3p
43	hsa-let-7e-5p	hsa-miR-324-5p	hsa-miR-193b-3p	hsa-miR-195-5p	hsa-miR-185-5p
44	hsa-miR-221-3p	hsa-miR-150-5p	hsa-miR-335-5p	hsa-miR-185-5p	hsa-miR-23a-3p
45	hsa-miR-26b-5p	hsa-miR-145-5p	hsa-miR-27a-3p	hsa-miR-143-3p	hsa-miR-107
46	hsa-miR-10b-5p	hsa-miR-103a-2-5p	hsa-miR-19b-3p	hsa-miR-186-5p	hsa-miR-199a-3p
47	hsa-miR-143-5p	hsa-miR-193a-3p	hsa-miR-148a-5p	hsa-miR-93-5p	hsa-miR-148b-3p
48	hsa-miR-628-3p	hsa-miR-484	hsa-miR-146a-5p	hsa-miR-140-3p	hsa-miR-140-3p
49	hsa-miR-423-5p	hsa-miR-130a-3p	hsa-miR-29c-3p	hsa-miR-375-3p	hsa-miR-17-5p
50	hsa-miR-519d-3p	hsa-miR-19a-3p	hsa-let-7e-5p	**hsa-let-7g-5p**	hsa-miR-19b-3p

**Table 2:** List of unique miRNAs in the top 50 of each of the five healthy sample types. For each miRNA, the individual rank is provided between brackets. In Supplementary Table S7, the unique physiological roles and pathway involvement to each set of specimen-specific miRNAs are shown. NAF: nipple aspirate fluid.

Samples	Number of miRNAs	miRNAs
**Breast tissue**	9	hsa-let-7c-5p (21) hsa-miR-100-5p (25) hsa-miR-199b-3p (31) hsa-miR-199a-5p (32) hsa-miR-497-5p (35) hsa-miR-939-5p (42) hsa-miR-143-5p (47) hsa-miR-628-3p (48) hsa-miR-519d-3p (50)
**NAF**	11	hsa-miR-203a-3p (2) hsa-miR-15a-5p (17) hsa-miR-34a-5p (21) hsa-miR-365a-3p (25) hsa-miR-652-3p (29) hsa-miR-210-3p (32) hsa-miR-361-5p (34) hsa-miR-29b-3p (35) hsa-miR-324-5p (43) hsa-miR-103a-2-5p (46) hsa-miR-193a-3p (47)
**Breast milk**	8	hsa-miR-200a-3p (9) hsa-miR-182-5p (20) hsa-miR-99b-5p (22) hsa-miR-429 (31) hsa-miR-10a-5p (37) hsa-miR-335-5p (44) hsa-miR-148a-5p (47) hsa-miR-29c-3p (49)
**Plasma**	7	hsa-miR-106a-5p (17) hsa-miR-222-3p (22) hsa-miR-142-3p (27) hsa-miR-192-5p (31) hsa-miR-425-5p (32) hsa-miR-106b-5p (35) hsa-miR-93-5p (47)
**Serum**	5	hsa-miR-142-5p (15) hsa-miR-423-3p (34) hsa-miR-16-2-3p (37) hsa-miR-107 (45) hsa-miR-148b-3p (47)

## Supplementary Materials

Supplementary materials can be found at https://www.mdpi.com/1422-0067/21/22/8466/s1. Supplementary Figure S1. Pearson correlation analysis between RT-qPCR profiling results and technical individual RT-qPCR assay validation (quality control) for three selected miRNAs showing high concordance. Supplementary Figure S2. Ranking range of the 20 most highly expressed miRNAs in breast tissue compared to the top 50 miRNAs from each study of each biosample: nipple aspirate fluid (NAF), breast milk, plasma and serum. Supplementary Figure S3. Physiological roles and pathway involvement of the top 20 NAF-derived miRNAs as retrieved from miRPathDB v2.0 using the Kyoto Encyclopedia of Genes and Genomes (KEGG) (A), WikiPathway (B) and Gene Ontology (GO) biological processes (C) databases. Supplementary Figure S4: Five-way Venn diagram showing the number of overlapping top 50 microRNAs across five sample types: breast tissue, NAF, breast milk, plasma and serum. Supplementary Methods S1. List of Advanced assay names present in the 754-miRNA panel used for NAF sample profiling. Supplementary Methods S2. Search syntaxes in PubMED used per biosample for the systematic literature search. Supplementary Methods S3. Inclusion and exclusion criteria used for article selection. Supplementary Methods S4. Flow Diagram: selection of articles for the physiological microRNA ranking based on breast tissue samples (A), breast milk samples (B), plasma samples (C) and serum samples (D). Supplementary Table S1. Individual expression levels, read counts or units of fluorescence units of the top 55–60 miRNAs per study that used breast tissue samples or breast tissue cell lines, breast milk, plasma, serum and NAF. Supplementary Table S2. Overall ranking of the top 50 miRNAs in studies that used breast tissue samples or breast tissue cell lines, breast milk, plasma and serum. Supplementary Table S3. Study subjects’ and sample characteristics of the studies included in the ranking. Supplementary Table S4. Overlap from the perspective of each sample as a reference and overall overlap. Supplementary Table S5. Proportion of immune-related miRNAs of the 50 most abundant miRNAs in NAF, breast milk, plasma and serum. Supplementary Table S6. Overview of the most relevant established mRNA targets and breast cancer related cellular processes of each top 20 NAF miRNA shown in Table 1. Supplementary Table S7. Unique physiological roles and pathway involvement to each set of specimen-specific miRNAs (Table 2) and for the 7 shared miRNAs (Table 1, bold).

## Abbreviations

DENSE	Dense tissue and Early breast Neoplasm Screening
GO	Gene Ontology
KEGG	Kyoto Encyclopedia of Genes and Genomes
miRNA	microRNA
NAF	Nipple Aspirate Fluid
PRISMA	Preferred Reporting Items for Systematic Reviews and Meta-Analyses
TCGA	The Cancer Genome Atlas
