# Supplementary material for: The Physiological MicroRNA Landscape in Nipple Aspirate Fluid: Differences and Similarities with Breast Tissue, Breast Milk, Plasma and Serum
Source: Int J Mol Sci. 2020 Nov 11;21(22):8466. doi: 10.3390/ijms21228466 (PMC7696615; doi:10.3390/ijms21228466)
Supplement: Supplementary file 1 [file ijms-21-08466-s001.zip › Supplementary Figures S1-4.docx]

**Supplementary Figure S1*.* Pearson correlation analysis between RT-qPCR profiling results and technical individual RT-qPCR assay validation (quality control) for three selected miRNAs showing high concordance.** Each dot represents a measurement of a NAF sample (n=41 NAF samples for hsa-miR-181a and hsa-miR-29a and n=40 NAF samples for hsa-miR-324).

**Supplementary Figure S2**. **Ranking range of the 20 most highly expressed miRNAs in breast tissue compared to the top 50 miRNAs from each study of each biosample: nipple aspirate fluid (NAF), breast milk, plasma and serum**. This figure shows that there is broad variation of miRNA ranking positions across studies, even within sample types. Each dot represents one study. Red horizontal lines indicate mean rank per biosample.

**Supplementary Figure S3. Physiological roles and pathway involvement of the top 20 NAF-derived miRNAs as retrieved from miRPathDB v2.0 (1) using the Kyoto Encyclopedia of Genes and Genomes (KEGG) (A), WikiPathway (B) and Gene Ontology (GO) biological processes (C) databases.** Stringent criteria for miRPathDB query included selection of data based on strong experimental evidence and with at least 10 significant miRNAs per pathway.

**A.
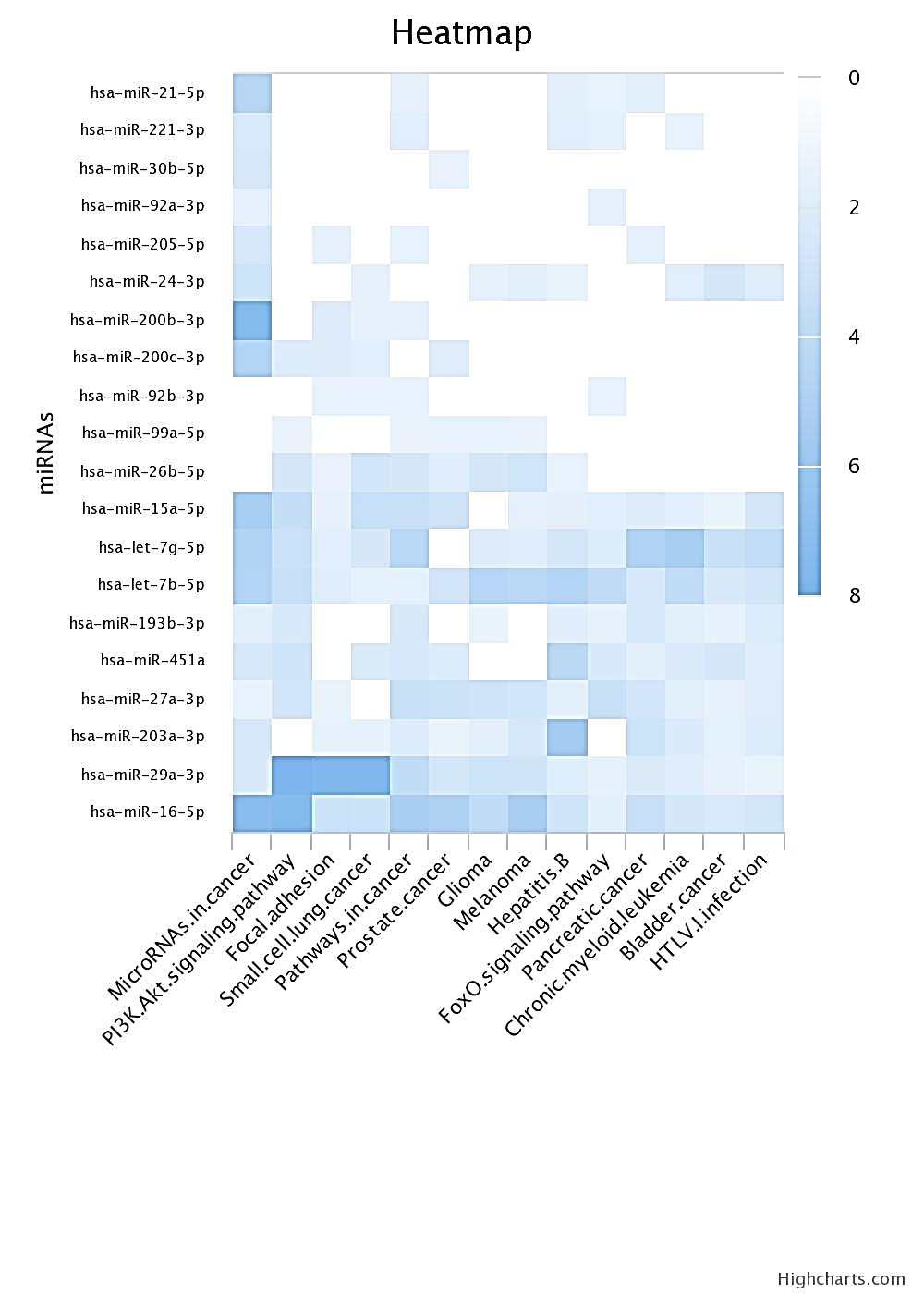
**

**B.**
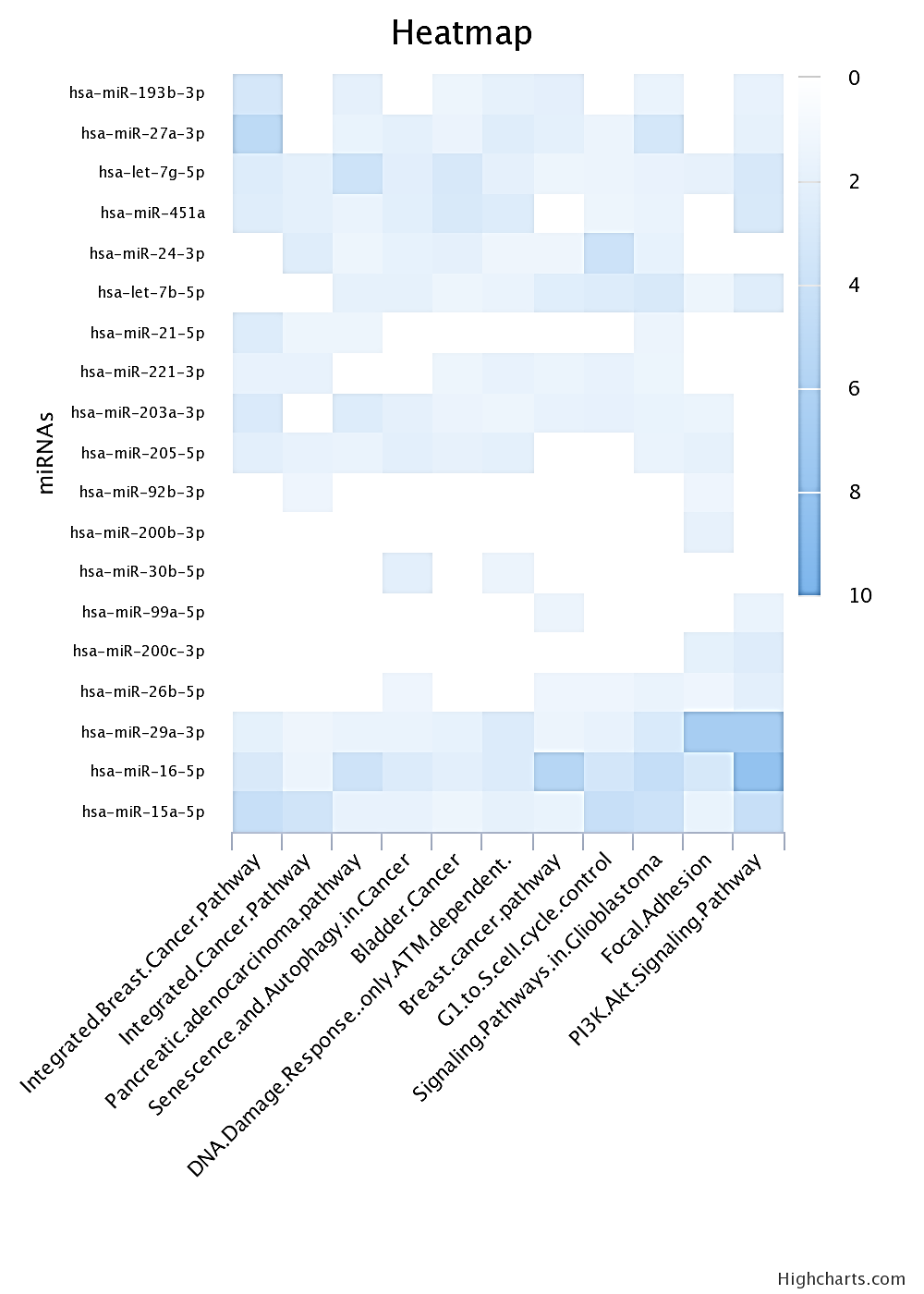


**C.
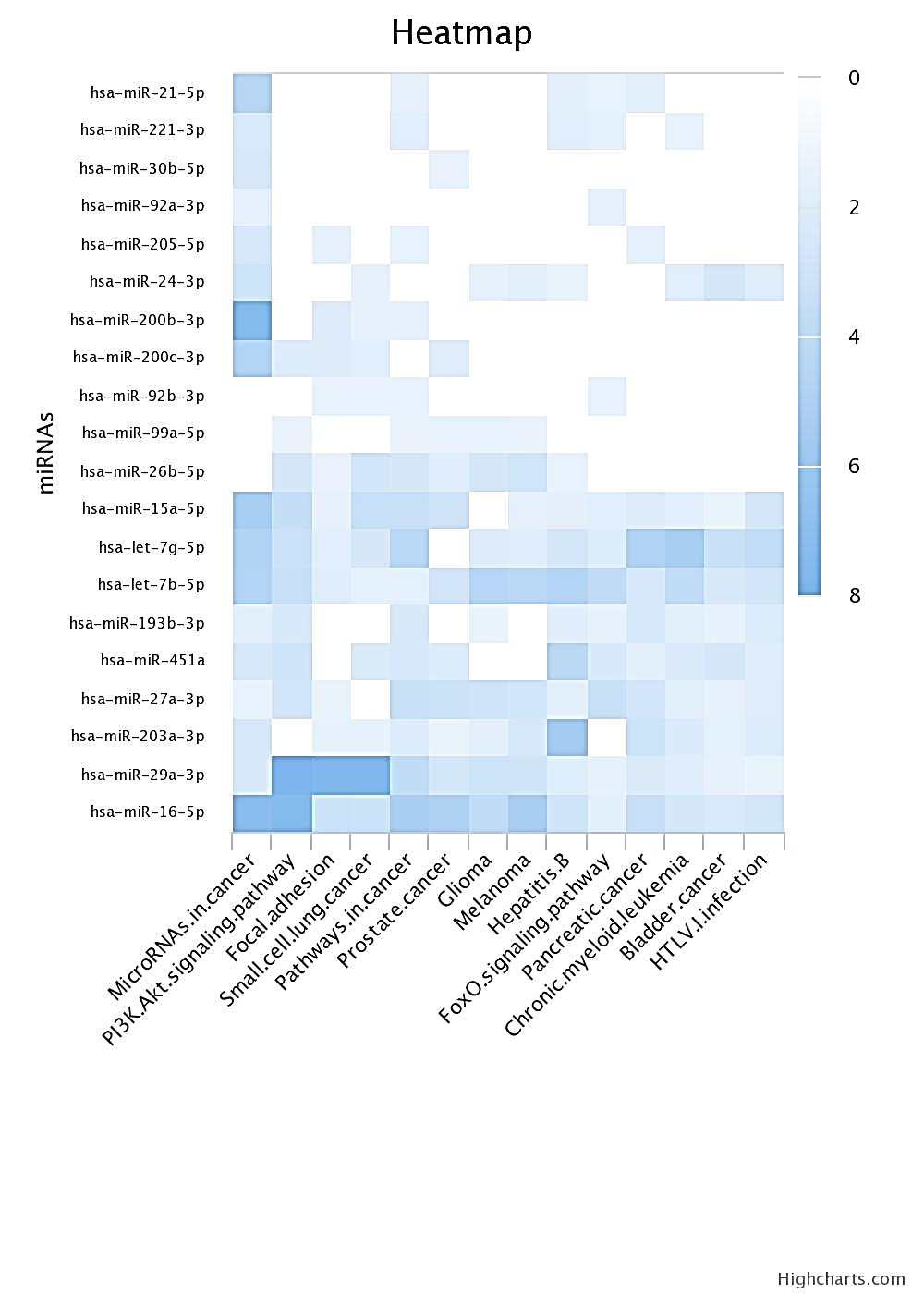
**

**Supplementary Figure A4. Five-way Venn diagram showing the number of overlapping top 50 microRNAs across five sample types: breast tissue, NAF, breast milk, plasma and serum.** See Supplementary Table A4F for the lists of microRNAs.


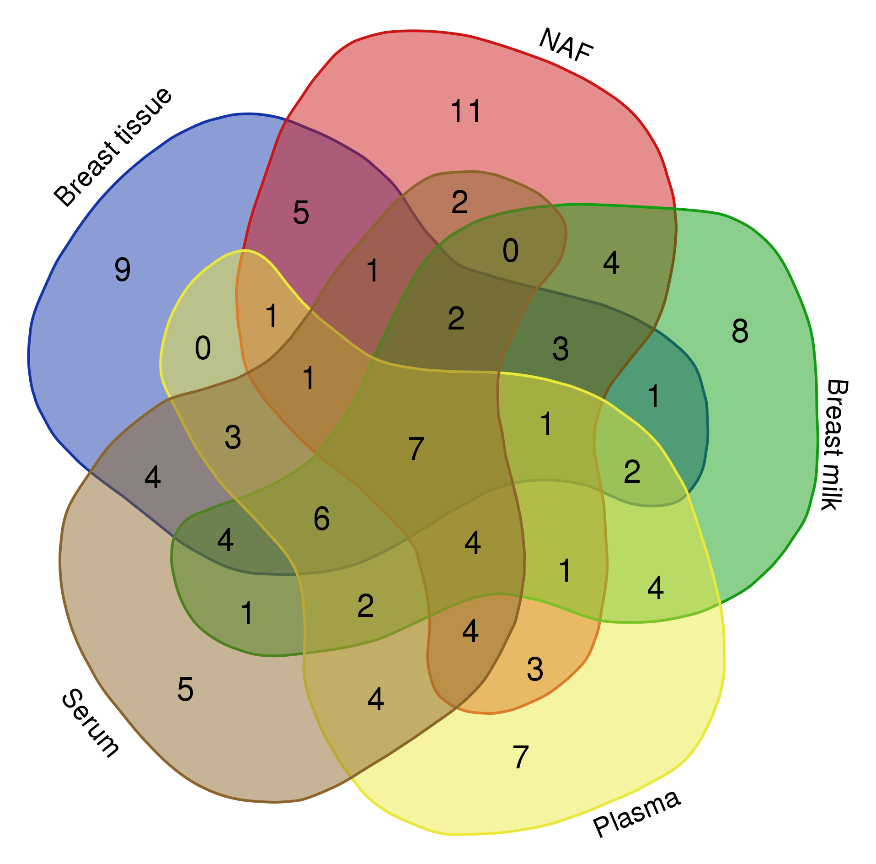


**References**

1. Backes C, Kehl T, Stockel D, Fehlmann T, Schneider L, Meese E, et al. miRPathDB: a new dictionary on microRNAs and target pathways. Nucleic Acids Res. 2017;45(D1):D90-D6.
