# Supplementary material for: The Physiological MicroRNA Landscape in Nipple Aspirate Fluid: Differences and Similarities with Breast Tissue, Breast Milk, Plasma and Serum
Source: Int J Mol Sci. 2020 Nov 11;21(22):8466. doi: 10.3390/ijms21228466 (PMC7696615; doi:10.3390/ijms21228466)
Supplement: Supplementary file 1 [file ijms-21-08466-s001.zip › Supplementary Methods S1-4.docx]

# Supplementary Methods S1. List of Advanced assay names present in the 754-miRNA panel used for NAF sample profiling. List adapted from Excel file available on the website of Thermo Fisher, titled “TaqMan OpenArray Human Advanced MicroRNA Panel Assay List” - version 1 layout. Publicly available at <https://www.thermofisher.com/order/catalog/product/A32710#/A32710>, accessed on 28 May 2020.

hsa-miR-409-5p hsa-miR-424-5p hsa-miR-30b-5p hsa-miR-29a-3p hsa-miR-485-3p hsa-miR-484 hsa-miR-380-3p hsa-miR-323b-5p hsa-miR-485-5p cel-miR-39-3p hsa-miR-449a hsa-miR-302b-3p hsa-miR-411-5p hsa-miR-324-5p hsa-miR-30c-5p hsa-miR-381-3p hsa-miR-376b-3p hsa-miR-296-3p hsa-miR-412-3p hsa-miR-429 hsa-miR-450a-5p hsa-miR-455-5p hsa-miR-302c-3p hsa-miR-204-5p ath-miR159a hsa-miR-145-5p hsa-miR-154-5p hsa-miR-132-3p hsa-miR-140-3p hsa-miR-27b-5p hsa-miR-15a-5p hsa-miR-146a-5p hsa-miR-148b-3p hsa-miR-127-3p hsa-miR-181a-5p hsa-miR-139-5p hsa-miR-142-5p hsa-miR-127-5p hsa-miR-130b-3p hsa-miR-182-5p hsa-miR-150-5p hsa-miR-129-5p hsa-miR-10a-3p hsa-miR-18b-5p hsa-miR-15b-5p hsa-miR-184 hsa-miR-18a-5p hsa-miR-548c-3p hsa-miR-582-5p hsa-miR-576-3p hsa-miR-548d-5p hsa-miR-16-5p hsa-miR-299-3p hsa-miR-28-5p hsa-miR-320a hsa-miR-376c-3p hsa-miR-423-5p hsa-miR-422a hsa-miR-377-3p hsa-miR-299-5p hsa-miR-214-3p cel-miR-39-3p hsa-miR-492 hsa-miR-483-5p hsa-let-7f-5p hsa-let-7e-5p hsa-miR-490-3p hsa-miR-219a-5p hsa-miR-23b-3p hsa-miR-30e-5p hsa-miR-17-5p hsa-let-7g-5p hsa-miR-216a-5p hsa-miR-219a-1-3p hsa-let-7i-5p hsa-miR-186-5p ath-miR159a hsa-miR-887-3p hsa-miR-744-5p hsa-miR-125a-3p hsa-miR-653-5p hsa-miR-22-5p hsa-miR-891a-5p hsa-miR-642a-5p hsa-miR-655-3p hsa-miR-99a-5p hsa-miR-99b-5p hsa-miR-651-5p hsa-miR-885-5p hsa-miR-636 hsa-miR-891b hsa-miR-875-3p hsa-miR-892a hsa-miR-146b-5p hsa-miR-24-2-5p hsa-miR-874-3p hsa-miR-876-3p hsa-miR-181d-5p hsa-miR-337-5p hsa-miR-326 hsa-miR-208b-3p hsa-miR-374b-5p hsa-miR-98-5p hsa-miR-16-5p hsa-miR-378a-5p hsa-miR-374a-3p hsa-miR-424-3p hsa-miR-409-3p hsa-miR-29a-5p hsa-miR-30d-3p hsa-miR-30e-3p hsa-miR-30a-5p hsa-miR-376a-5p cel-miR-39-3p hsa-miR-302b-5p hsa-miR-302d-3p hsa-miR-30c-2-3p hsa-miR-31-3p hsa-miR-431-3p hsa-miR-302c-5p hsa-miR-218-2-3p hsa-miR-432-5p hsa-miR-138-1-3p hsa-miR-302d-5p hsa-let-7e-3p hsa-miR-488-5p hsa-miR-23a-5p hsa-miR-1260a ath-miR159a hsa-miR-190b hsa-miR-1227-3p hsa-miR-1256 hsa-miR-1250-5p hsa-miR-27a-5p hsa-miR-1205 hsa-miR-1233-3p hsa-miR-1204 hsa-miR-126-5p hsa-miR-1247-5p hsa-miR-1263 hsa-miR-1249-3p hsa-miR-1206 hsa-miR-125b-2-3p hsa-miR-1245a hsa-miR-637 hsa-miR-1244 hsa-miR-34a-3p hsa-miR-1262 hsa-miR-551b-5p hsa-miR-1238-3p hsa-miR-656-3p hsa-miR-559 hsa-miR-584-5p hsa-miR-30a-3p hsa-miR-1324 hsa-miR-16-5p hsa-let-7f-2-3p hsa-miR-106b-3p hsa-miR-23b-5p hsa-miR-221-5p hsa-miR-1179 hsa-miR-100-3p hsa-miR-181a-3p cel-miR-39-3p hsa-miR-1182 hsa-let-7a-3p hsa-miR-193b-5p hsa-miR-105-3p hsa-miR-26b-3p hsa-miR-101-5p hsa-miR-19b-1-5p hsa-miR-1183 hsa-miR-708-3p hsa-miR-744-3p ath-miR159a hsa-miR-638 hsa-miR-661 hsa-miR-650 hsa-miR-7-2-3p hsa-miR-647 hsa-miR-335-3p hsa-miR-662 hsa-miR-659-3p hsa-miR-675-5p hsa-miR-934 hsa-miR-9-3p hsa-miR-644a hsa-miR-922 hsa-miR-639 hsa-miR-939-5p hsa-miR-549a hsa-miR-92b-5p hsa-miR-558 hsa-miR-580-3p hsa-miR-130a-5p hsa-miR-16-5p hsa-miR-100-5p hsa-let-7d-5p hsa-miR-210-3p hsa-miR-217 hsa-miR-22-3p hsa-miR-103a-3p hsa-miR-365a-3p_hsa-miR-365b-3p hsa-miR-2110 hsa-miR-224-5p cel-miR-39-3p hsa-miR-361-5p hsa-miR-212-3p hsa-miR-192-5p hsa-let-7c-5p hsa-miR-342-5p hsa-miR-346 hsa-miR-340-5p hsa-miR-345-5p hsa-miR-509-5p hsa-miR-198 hsa-miR-203a-3p hsa-miR-200b-3p hsa-miR-202-3p hsa-let-7b-5p ath-miR159a hsa-miR-499a-3p hsa-miR-494-3p hsa-miR-504-5p hsa-miR-515-3p hsa-miR-769-5p hsa-miR-517c-3p hsa-miR-488-3p hsa-miR-518b hsa-miR-503-5p hsa-miR-140-5p hsa-miR-509-3-5p hsa-miR-502-5p hsa-miR-516a-5p hsa-miR-508-3p hsa-miR-518a-3p hsa-miR-629-5p hsa-miR-598-3p hsa-miR-943 hsa-miR-597-5p hsa-miR-615-5p hsa-miR-624-3p hsa-miR-628-5p hsa-miR-561-3p hsa-miR-324-3p hsa-miR-95-3p hsa-miR-496 hsa-miR-16-5p hsa-miR-370-3p hsa-miR-328-3p hsa-miR-197-3p hsa-miR-338-3p hsa-miR-342-3p hsa-miR-193b-3p hsa-miR-199b-5p hsa-miR-20b-5p hsa-miR-331-5p cel-miR-39-3p hsa-miR-33b-5p hsa-miR-369-3p hsa-miR-369-5p hsa-miR-208a-3p hsa-miR-542-5p hsa-miR-508-5p hsa-miR-139-3p hsa-miR-517a-3p_hsa-miR-517b-3p hsa-miR-520a-3p hsa-miR-199a-5p hsa-miR-526b-5p hsa-miR-196b-5p hsa-miR-519e-3p hsa-miR-525-3p ath-miR159a hsa-miR-518f-3p hsa-miR-542-3p hsa-miR-518d-3p hsa-miR-455-3p hsa-miR-1283 hsa-miR-576-5p hsa-miR-200c-3p hsa-miR-525-5p hsa-miR-524-5p hsa-miR-93-5p hsa-miR-520g-3p hsa-miR-518e-3p hsa-miR-520f-3p hsa-miR-301b-3p hsa-miR-520e hsa-miR-450b-5p hsa-miR-570-3p hsa-miR-1267 hsa-miR-544a hsa-miR-548a-5p hsa-miR-548d-3p hsa-miR-26b-5p hsa-miR-129-2-3p hsa-miR-487b-3p hsa-miR-491-5p hsa-miR-499a-5p hsa-miR-16-5p hsa-miR-195-3p hsa-miR-34b-3p hsa-miR-337-3p hsa-miR-577 hsa-miR-33a-3p hsa-miR-645 hsa-miR-363-5p hsa-miR-20a-3p hsa-miR-19a-5p cel-miR-39-3p hsa-miR-135b-3p hsa-miR-145-3p hsa-miR-1290 hsa-miR-361-3p hsa-miR-1296-5p hsa-miR-191-3p hsa-miR-154-3p hsa-miR-151a-5p hsa-miR-34c-3p hsa-miR-151a-3p hsa-miR-1265 hsa-miR-181a-2-3p hsa-miR-182-3p hsa-miR-802 ath-miR159a hsa-miR-92a-1-5p hsa-miR-767-3p hsa-miR-1278 hsa-miR-888-3p hsa-miR-1243 hsa-miR-93-3p hsa-miR-941 hsa-miR-588 hsa-miR-513c-5p hsa-miR-603 hsa-miR-937-3p hsa-miR-617 hsa-miR-635 hsa-miR-614 hsa-miR-630 hsa-miR-586 hsa-miR-590-3p hsa-miR-604 hsa-miR-10b-3p hsa-miR-517-5p hsa-miR-592 hsa-miR-613 hsa-miR-557 hsa-miR-548p hsa-miR-601 hsa-miR-553 hsa-miR-16-5p hsa-miR-16-1-3p hsa-miR-138-2-3p hsa-miR-148b-5p hsa-miR-569 hsa-miR-144-5p hsa-miR-203b-5p hsa-miR-1276 cel-miR-39-3p hsa-miR-188-5p hsa-miR-1304-5p hsa-miR-151b hsa-miR-515-5p hsa-miR-1275 hsa-miR-183-3p hsa-miR-1270 hsa-miR-1236-3p hsa-miR-181c-3p hsa-miR-595 hsa-miR-18a-3p hsa-miR-609 ath-miR159a hsa-miR-620 hsa-miR-585-3p hsa-miR-1292-5p hsa-miR-548e-3p hsa-miR-548n hsa-miR-623 hsa-miR-518f-5p hsa-miR-378a-3p hsa-miR-524-3p hsa-miR-626 hsa-miR-548g-3p hsa-miR-513b-5p hsa-miR-600 hsa-miR-548i hsa-miR-548l hsa-miR-550a-5p hsa-miR-520d-3p hsa-miR-500a-3p hsa-miR-519e-5p hsa-miR-571 hsa-let-7d-3p hsa-miR-518e-5p_hsa-miR-519a-5p_hsa-miR-519b-5p_hsa-miR-519c-5p_hsa-miR-522-5p_hsa-miR-523-5p hsa-miR-7-1-3p hsa-miR-16-5p hsa-miR-126-3p hsa-miR-433-3p hsa-miR-452-5p hsa-miR-425-5p hsa-miR-302a-3p hsa-miR-375 hsa-miR-323a-3p hsa-miR-448 hsa-miR-376a-3p cel-miR-39-3p hsa-miR-431-5p hsa-miR-29b-3p hsa-miR-454-3p hsa-miR-31-5p hsa-miR-486-5p hsa-miR-513a-5p hsa-miR-382-5p hsa-miR-449b-5p hsa-miR-141-3p hsa-miR-32-5p hsa-miR-298 hsa-miR-486-3p hsa-miR-29c-3p hsa-miR-135a-5p ath-miR159a hsa-miR-146b-3p hsa-miR-147b hsa-miR-128-3p hsa-miR-130a-3p hsa-miR-222-5p hsa-miR-143-3p hsa-miR-142-3p hsa-miR-138-5p hsa-miR-1264 hsa-miR-548b-5p hsa-miR-147a hsa-miR-135b-5p hsa-miR-183-5p hsa-miR-188-3p hsa-miR-133a-3p hsa-miR-152-3p hsa-miR-551b-3p hsa-miR-150-3p hsa-miR-185-5p hsa-miR-191-5p hsa-miR-133b hsa-miR-190a-5p hsa-let-7a-5p hsa-miR-211-5p hsa-miR-579-3p hsa-miR-556-5p hsa-miR-16-5p hsa-miR-410-3p hsa-miR-487a-3p hsa-miR-28-3p hsa-miR-383-5p hsa-miR-107 hsa-miR-384 hsa-miR-450b-3p hsa-miR-26a-5p hsa-miR-423-3p cel-miR-39-3p hsa-miR-10a-5p hsa-miR-489-3p hsa-miR-222-3p hsa-miR-105-5p hsa-miR-221-3p hsa-miR-125a-5p hsa-miR-10b-5p hsa-miR-301a-3p hsa-miR-181c-5p hsa-miR-491-3p hsa-miR-219a-2-3p hsa-miR-215-5p hsa-miR-27b-3p hsa-miR-654-5p ath-miR159a hsa-miR-106b-5p hsa-miR-103a-2-5p hsa-miR-125b-5p hsa-miR-660-5p hsa-miR-338-5p hsa-miR-708-5p hsa-miR-671-3p hsa-miR-153-3p hsa-miR-21-5p hsa-miR-654-3p hsa-miR-34a-5p hsa-miR-888-5p hsa-miR-652-3p hsa-miR-92b-3p hsa-miR-885-3p hsa-miR-873-5p hsa-miR-122-5p hsa-miR-643 hsa-miR-876-5p hsa-miR-181b-5p hsa-miR-889-3p hsa-miR-92a-3p hsa-miR-758-3p hsa-miR-890 hsa-miR-205-5p hsa-miR-187-3p hsa-miR-16-5p hsa-miR-374b-3p hsa-miR-377-5p hsa-miR-452-3p hsa-miR-567 hsa-miR-302a-5p hsa-miR-30b-3p hsa-miR-425-3p hsa-miR-770-5p hsa-miR-29b-2-5p cel-miR-39-3p hsa-miR-30c-1-3p hsa-miR-432-3p hsa-miR-32-3p hsa-miR-454-5p hsa-miR-30d-5p hsa-miR-380-5p hsa-miR-25-5p hsa-miR-320b hsa-miR-106a-3p hsa-miR-29b-1-5p hsa-miR-214-5p hsa-miR-1184 hsa-let-7f-1-3p hsa-miR-483-3p ath-miR159a hsa-miR-18b-3p hsa-miR-125b-1-3p hsa-miR-1248 hsa-miR-155-3p hsa-miR-875-5p hsa-miR-1252-5p hsa-miR-1254 hsa-miR-1255b-5p hsa-miR-15b-3p hsa-miR-1226-5p hsa-miR-124-5p hsa-miR-1225-3p hsa-miR-1228-5p hsa-miR-1251-5p hsa-miR-122-3p hsa-miR-1825 hsa-miR-641 hsa-miR-767-5p hsa-miR-1255a hsa-miR-649 hsa-miR-1224-3p hsa-miR-631 hsa-miR-1208 hsa-miR-564 hsa-miR-581 hsa-miR-555 hsa-miR-16-5p hsa-miR-21-3p hsa-miR-1200 hsa-miR-26a-2-3p hsa-miR-578 hsa-let-7i-3p hsa-let-7g-3p hsa-miR-1180-3p hsa-miR-7-5p cel-miR-39-3p hsa-let-7b-3p hsa-miR-26a-1-3p hsa-miR-223-5p hsa-miR-24-1-5p hsa-miR-1197 hsa-miR-202-5p hsa-miR-218-1-3p hsa-miR-196a-3p hsa-miR-1178-3p hsa-miR-143-5p hsa-miR-367-5p hsa-miR-633 ath-miR159a hsa-miR-646 hsa-miR-665 hsa-miR-657 hsa-miR-634 hsa-miR-130b-5p hsa-miR-658 hsa-miR-648 hsa-miR-668-3p hsa-miR-663b hsa-miR-196b-3p hsa-miR-766-3p hsa-miR-640 hsa-miR-924 hsa-miR-92a-2-5p hsa-miR-935 hsa-miR-1298-5p hsa-miR-944 hsa-miR-938 hsa-miR-892b hsa-miR-563 hsa-miR-583 hsa-miR-573 hsa-miR-554 hsa-miR-16-5p hsa-miR-296-5p hsa-miR-1-3p hsa-miR-216b-5p hsa-miR-223-3p hsa-miR-329-3p hsa-miR-24-3p hsa-miR-101-3p hsa-miR-218-5p hsa-miR-25-3p cel-miR-39-3p hsa-miR-106a-5p hsa-miR-27a-3p hsa-miR-199a-3p_hsa-miR-199b-3p hsa-miR-186-3p hsa-miR-373-3p hsa-miR-200a-3p hsa-miR-374a-5p hsa-miR-371a-3p hsa-miR-325 hsa-miR-362-5p hsa-miR-516b-5p hsa-miR-19b-3p hsa-miR-331-3p hsa-miR-493-3p ath-miR159a hsa-miR-501-3p hsa-miR-512-5p hsa-miR-507 hsa-miR-505-3p hsa-miR-608 hsa-miR-518c-3p hsa-miR-501-5p hsa-miR-518d-5p_hsa-miR-520c-5p_hsa-miR-526a hsa-miR-506-3p hsa-miR-500a-5p hsa-miR-512-3p hsa-miR-618 hsa-miR-421 hsa-miR-510-5p hsa-miR-590-5p hsa-miR-589-5p hsa-miR-625-5p hsa-miR-516a-3p_hsa-miR-516b-3p hsa-miR-616-3p hsa-miR-96-5p hsa-miR-627-5p hsa-miR-502-3p hsa-miR-582-3p hsa-miR-379-5p hsa-miR-124-3p hsa-miR-23a-3p hsa-miR-16-5p hsa-miR-20a-5p hsa-miR-137 hsa-miR-495-3p hsa-miR-363-3p hsa-miR-148a-3p hsa-miR-330-3p hsa-miR-367-3p hsa-miR-335-5p hsa-miR-339-3p cel-miR-39-3p hsa-miR-34c-5p hsa-miR-195-5p hsa-miR-372-3p hsa-miR-149-5p hsa-miR-193a-5p hsa-miR-136-5p hsa-miR-362-3p hsa-miR-339-5p hsa-miR-330-5p hsa-miR-519d-3p hsa-miR-19a-3p hsa-miR-193a-3p hsa-miR-194-5p hsa-miR-511-5p ath-miR159a hsa-miR-518a-5p_hsa-miR-527 hsa-miR-520d-5p hsa-miR-155-5p hsa-miR-541-3p hsa-miR-545-5p hsa-miR-523-3p hsa-miR-539-5p hsa-miR-532-3p hsa-miR-519c-3p hsa-miR-545-3p hsa-miR-520b hsa-miR-519a-3p hsa-miR-522-3p hsa-miR-520a-5p hsa-miR-521 hsa-miR-574-3p hsa-miR-532-5p hsa-miR-548h-5p hsa-miR-548b-3p hsa-miR-548am-5p_hsa-miR-548c-5p_hsa-miR-548o-5p hsa-miR-556-3p hsa-miR-548a-3p hsa-miR-451a hsa-miR-9-5p hsa-miR-615-3p hsa-miR-134-5p hsa-miR-16-5p hsa-miR-200a-5p hsa-miR-200b-5p hsa-miR-550a-3p hsa-miR-192-3p hsa-miR-200c-5p hsa-miR-20b-3p hsa-miR-664a-3p hsa-miR-136-3p hsa-miR-141-5p cel-miR-39-3p hsa-miR-194-3p hsa-miR-206 hsa-miR-552-3p hsa-miR-340-3p hsa-miR-33a-5p hsa-miR-144-3p hsa-miR-149-3p hsa-miR-1294 hsa-miR-148a-5p hsa-miR-942-5p hsa-miR-1302 hsa-miR-1291 hsa-miR-16-2-3p hsa-miR-921 ath-miR159a hsa-miR-933 hsa-miR-1284 hsa-miR-411-3p hsa-miR-920 hsa-miR-765 hsa-miR-936 hsa-miR-497-3p hsa-miR-593-3p hsa-miR-34b-5p hsa-miR-607 hsa-miR-606 hsa-miR-622 hsa-miR-518c-5p hsa-miR-628-3p hsa-miR-587 hsa-miR-589-3p hsa-miR-593-5p hsa-miR-769-3p hsa-miR-1286 hsa-miR-596 hsa-miR-621 hsa-miR-605-5p hsa-miR-562 hsa-miR-551a hsa-miR-572 hsa-miR-566 hsa-miR-16-5p hsa-miR-99b-3p hsa-miR-17-3p hsa-miR-1288-3p hsa-miR-1271-5p hsa-miR-1269a hsa-miR-129-1-3p hsa-miR-1301-3p hsa-miR-185-3p hsa-miR-1272 cel-miR-39-3p hsa-miR-146a-3p hsa-miR-196a-5p hsa-miR-1285-3p hsa-miR-1293 hsa-miR-15a-3p hsa-miR-1289 hsa-miR-1253 hsa-miR-1303 hsa-miR-1203 hsa-miR-1282 hsa-miR-1257 hsa-miR-616-5p ath-miR159a hsa-miR-624-5p hsa-miR-591 hsa-miR-629-3p hsa-miR-599 hsa-miR-625-3p hsa-miR-520h hsa-miR-497-5p hsa-miR-543 hsa-miR-505-5p hsa-miR-548j-5p hsa-miR-548m hsa-miR-541-5p hsa-miR-520c-3p hsa-miR-132-5p hsa-miR-519b-3p hsa-miR-548k hsa-miR-575 hsa-miR-96-3p hsa-miR-99a-3p hsa-miR-29c-5p hsa-miR-16-5p

**Supplementary Methods S2. Search syntaxes in PubMED used per biosample for systematic literature search**

**Breast milk**

("microRNAs" [MeSH Terms] OR microrna*[Title/Abstract] OR micro rna [Title/Abstract] OR micro rnas [Title/Abstract] OR mirna* [Title/Abstract]) AND ("milk, human"[MeSH Terms] OR breast milk [Title/Abstract] OR breastmilk [Title/Abstract] OR human milk[Title/Abstract]) AND ("microarray analysis"[MeSH Terms] OR microarray* [Title/Abstract] OR array* [Title/Abstract] OR profil* [Title/Abstract] OR sequenc* [Title/Abstract])

**Tissue**

("microRNAs" [MeSH Terms] OR microrna* [Title/Abstract] OR micro rna [Title/Abstract] OR micro rnas [Title/Abstract] OR mirna* [Title/Abstract]) AND ("mammary glands, human" [MeSH Terms] OR mammary gland* [Title/Abstract] OR breast tissue* [Title/Abstract] OR breast connective tissue*[Title/Abstract] OR mammary tissue[Title/Abstract]) AND ("healthy volunteers"[MeSH Terms] OR healthy volunteer*[Title/Abstract] OR healthy participant*[Title/Abstract] OR healthy subject*[Title/Abstract] OR human volunteer*[Title/Abstract] OR normal volunteer*[Title/Abstract] OR control*[Title/Abstract] OR healthy women[Title/Abstract]) AND ("microarray analysis"[MeSH Terms] OR microarray*[Title/Abstract] OR array*[Title/Abstract] OR profil* [Title/Abstract] OR sequenc*[Title/Abstract])

**Plasma^a^**

("microRNAs" [MeSH Terms] OR microrna* [Title/Abstract] OR mirna* [Title/Abstract]) AND ("plasma"[MeSH Terms] OR plasma[Title/Abstract]) AND (women[Title/Abstract]) AND ("microarray analysis"[MeSH Terms] OR microarray*[Title/Abstract] OR array*[Title/Abstract] OR profil*[Title/Abstract] OR sequenc*)

**Serum^a^**

("microRNAs" [MeSH Terms] OR microrna* [Title/Abstract] OR mirna* [Title/Abstract]) AND ("serum"[MeSH Terms] OR serum[Title/Abstract]) AND (women[Title/Abstract]) AND ("microarray analysis"[MeSH Terms] OR microarray*[Title/Abstract] OR array*[Title/Abstract] OR profil*[Title/Abstract] OR sequenc*)

a. For plasma and serum, we decided to only search for articles that performed miRNA non-targeted analysis, to ensure that a ranking based on data from women was obtained.

**Supplementary Methods S3. Inclusion and exclusion criteria used for article selection**

**Inclusion criteria**

Studies were included if they met the following inclusion criteria:

(1) samples were collected from healthy women,

(2) microRNA expression analysis was performed in breast tissue, breast milk, plasma or serum

(3) microRNA relative quantitative measure presentation based on non-targeted techniques such as profiling, sequencing and microarray

(4) -3p and -5p arms of microRNAs mentioned (if applicable)

**Exclusion criteria:**

Studies were excluded if:

(1) the format was a review, conference abstract, comment, perspective or book chapter,

(2) not written in English,

(3) full text was not available,

(4) samples were from animals,

(5) miRNA analysis was performed in cell lines^a^

(6) samples from women with benign disease were defined as the control samples,

(7) tissue samples originated from healthy adjacent tumor area,

(8) samples were from pregnant women

(9) samples were from women with an explicit condition or complaint,

(10) blood samples, without explicit differentiation between serum or plasma fraction and hence separated data,

(11) samples from men which data could not be separated from that of women,

(12) individual quantitative measures (Ct values or read counts) for the analyzed miRNAs were absent either in the article, supplementary data or data repositories,

(13) less than 50 miRNAs were analyzed.

a. Given the low number of articles that allowed generating a ranking for breast tissue, an exception was made for studies using breast cell lines that resemble epithelial cells.

**Supplementary Methods S4A. Flow Diagram: selection of articles for the physiological microRNA ranking based on breast tissue samples**


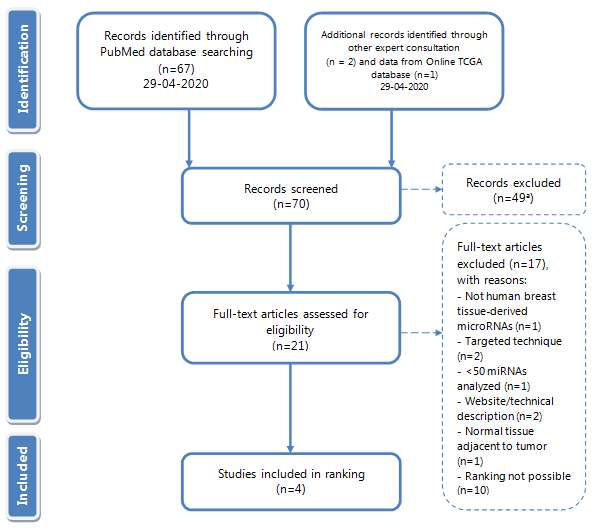


a. This includes exclusion of the online The Cancer Genome Atlas (TCGA) database given that ‘normal breast tissue’ samples referred to samples adjacent to tumor tissue.

Based on: Moher D, Liberati A, Tetzlaff J, Altman DG, The PRISMA Group (2009). Preferred Reporting Items for Systematic Reviews and Meta-Analyses: The PRISMA Statement. PLoS Med 6(7): e1000097. doi:10.1371/journal.pmed1000097

For more information, visit [www.prisma-statement.org](http://www.consort-statement.org/).

**Supplementary Methods S4B. Flow Diagram: selection of articles for the physiological microRNA ranking based on breast milk samples**


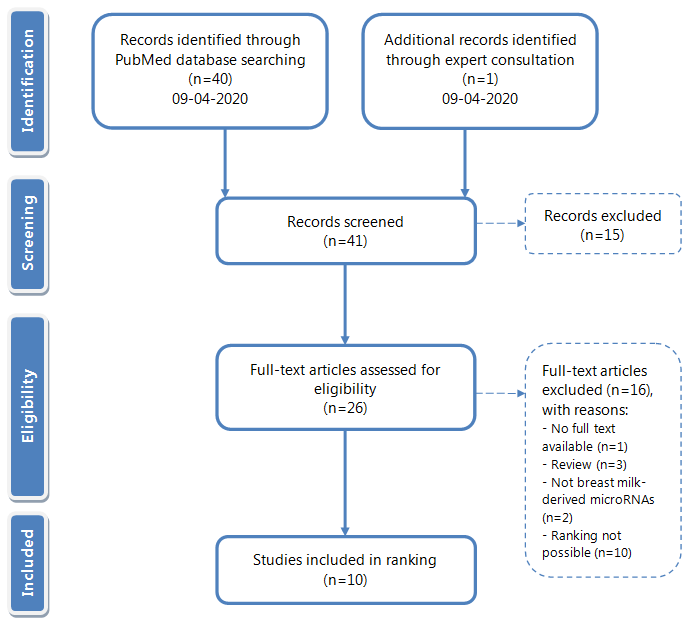


Based on: Moher D, Liberati A, Tetzlaff J, Altman DG, The PRISMA Group (2009). Preferred Reporting Items for Systematic Reviews and Meta-Analyses: The PRISMA Statement. PLoS Med 6(7): e1000097. doi:10.1371/journal.pmed1000097

For more information, visit [www.prisma-statement.org](http://www.consort-statement.org/).

**Supplementary Methods S4C. Flow Diagram: selection of articles for the physiological microRNA ranking based on plasma samples**


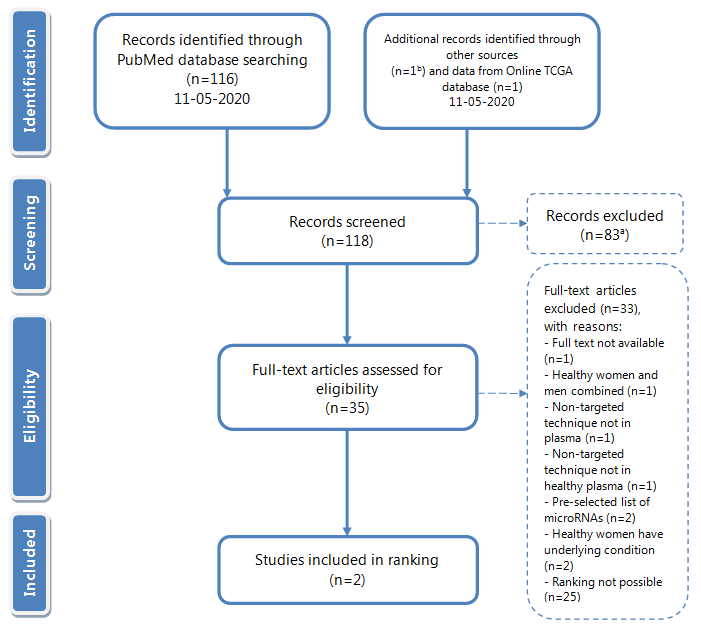


1. This includes exclusion of the online The Cancer Genome Atlas (TCGA) database given that ‘plasma’ was not specified for microRNA data derived from blood samples.
2. Article added from “breast milk” search

Based on: Moher D, Liberati A, Tetzlaff J, Altman DG, The PRISMA Group (2009). Preferred Reporting Items for Systematic Reviews and Meta-Analyses: The PRISMA Statement. PLoS Med 6(7): e1000097. doi:10.1371/journal.pmed1000097

For more information, visit [www.prisma-statement.org](http://www.consort-statement.org/).

**Supplementary Methods S4D. Flow Diagram: selection of articles for the physiological microRNA ranking based on serum samples**


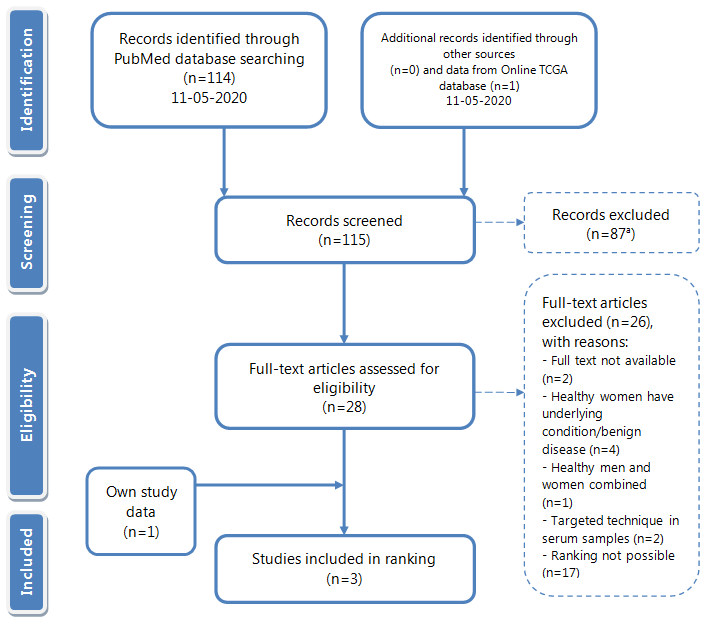


a. This includes exclusion of the online The Cancer Genome Atlas (TCGA) database given that ‘serum’ was not specified for microRNA data derived from blood samples.

Based on: Moher D, Liberati A, Tetzlaff J, Altman DG, The PRISMA Group (2009). Preferred Reporting Items for Systematic Reviews and Meta-Analyses: The PRISMA Statement. PLoS Med 6(7): e1000097. doi:10.1371/journal.pmed1000097

For more information, visit [www.prisma-statement.org](http://www.consort-statement.org/).
