# Supplementary material for: The Physiological MicroRNA Landscape in Nipple Aspirate Fluid: Differences and Similarities with Breast Tissue, Breast Milk, Plasma and Serum
Source: Int J Mol Sci. 2020 Nov 11;21(22):8466. doi: 10.3390/ijms21228466 (PMC7696615; doi:10.3390/ijms21228466)
Supplement: Supplementary file 1 [file ijms-21-08466-s001.zip › Supplementary Table S6.docx]

**Supplementary Table S6. Overview of the most relevant established mRNA targets and breast cancer related cellular processes of each top 20 NAF miRNA shown in Table 1.** Messenger RNAs targeted by six or more top 20 miRNAs are indicated in bold. TAM: tumor associated macrophage; CSC: cancer stem cell; EMT: epithelial to mesenchymal transition. *same cluster: miR 27a~24-2; ^#^same cluster: miR 15a~16-1.

|  | **Tumor suppressor or oncogenic role** | **Established targets** | **Processes** | **References** |
| --- | --- | --- | --- | --- |
| **hsa-miR-205-5p** | Tumor suppressor | ERBB2/3, **ZEB1/2**, **VEGFA**, E2F1, HMGB1/3, **PTEN**, MED1, SMAD1/2/4, **BCL2**/6 | EMT; proliferation; stemness; expansion of the progenitor-cell population | (1-8) |
| **hsa-miR-203a-3p** | Tumor suppressor | **ZEB2**, TP63, **BMI1**, SNAI1/2, SOCS3, SRC, BIRC5, CDK6, SMAD4, **VEGFA**, MMP10, ABL1 | EMT; cell shape; matrix adhesion; migration/invasion; stemness | (9-13) |
| **hsa-miR-26b-5p** | Tumor suppressor | PTGS2, GATA4, EZH2, COL1A2, LARP1, CDK6, CCNE1, TAB1, EPHA2, KPNA2, PGR, **PTEN** | apoptosis; proliferation | (14-17) |
| **hsa-miR-221-3p** | Oncogenic | CDKN1B/c, BCL2L11, DDIT4, KIT, ETS1, ESR1, MDM2, **ZEB2**, PIK3R1, **PTEN**, FOXO3, BNIP3, PARP | apoptosis; proliferation | (18, 19) |
| **hsa-let-7b-5p** | Tumor suppressor | HMGA1/2, **CCND1/2**, CDC34, IGF1R, AGO1, PRDM1, CDK6, CDC25A, IGF2BP1/2 | migration/invasion | (20, 21) |
| **hsa-miR-27a-3p*** | Oncogenic | PHB, FOXO1, SPRY2, ZBTB10, SMAD2/4/5, CDC27, SFRP1, TP53, FBXW7, SP1, EGFR, MET, **CCND1** | proliferation; apoptosis; EMT; migration/invasion; angiogenesis; TAM polarization | (22-29) |
| **hsa-miR-451a** | Tumor suppressor | MIF, ABCB1, CAB39, STAT3, IL6/R, MYC, AKT1, **BCL2**, MAP3K1, TSC1 | proliferation; migration/invasion; apoptosis; autophagy | (30-33) |
| **hsa-miR-92a-3p** | Tumor suppressor | BMPR2, HIPK1, KLF2, ITGA5, TP63, STAT3, **PTEN**, FBXW7, MAP2K4 | migration/invasion | (14, 34) |
| **hsa-miR-16-5p^#^** | Tumor supressor | CCNE1, FGF2, ARL2, **BCL2**, HMGA1, CDK6, CCND1, **VEGFA**, RECK, PRDM4, PPM1D, WEE1, CHEK1, **BMI1** | proliferation; apoptosis; stress response; CSC growth | (35-39) |
| **hsa-let-7g-5p** | Tumor suppressor | HMGA2, IGF2BP1, COL1A2, MYC, CDKN2A, BCL2L1, AGO1, THBS1, AKT2, FN1, SMAD2, TGFBR1, FOXC2, **BMI1** | proliferation; migration/invasion | (21, 40-42) |
| **hsa-miR-24-3p*** | Oncogenic | CDKN1B, FEN1, CDK4, CCNA2, AURKB, MYC, E2F2, DHFR, HNF4A, SP1, BRCA1, POLD1,H2AFX, **CCND1**, CDK1 | proliferation; apoptosis | (43-46) |
| **hsa-miR-200c-3p** | Tumor suppressor | **ZEB1/2**, TUBB3, **BMI1**, FN1, RNF2, DUSP1, ZNF217, KRAS, CDK2, SP1, XIAP, **BCL2**, **PTEN**, **VEGFA** | EMT; migration/invasion; stemness | (5, 47-56) |
| **hsa-miR-200b-3p** | Tumor suppressor | **ZEB1/2**, FN1, **BMI1**, RNF2, ETS1, GATA4, WNT1, CDKN1B, **VEGFA**, XIAP, **BCL2**, CREB1, MYB, SP1 | proliferation; migration/invasion; stemness | (5, 56-61) |
| **hsa-miR-99a-5p** | Tumor suppressor | MTOR, SMARCA5, IGF1R, FGFR3, AGO2, CAPNS1, HOXA1, CDC25A | EMT; migration/invasion; proliferation; apoptosis | (62-67) |
| **hsa-miR-21-5p** | Oncogenic | RASGRP1, CDC25A, BTG2, PDCD4, SERPINB5, **BCL2**, RPS7, TIMP3, SOX5, SP1, RECK, TGFBR2/3, **PTEN**, **VEGFA** | proliferation; apoptosis, EMT; migration/invasion | (68-73) |
| **hsa-miR-92b-3p** | Tumor suppressor/Oncogenic | SLC15A1, DAB2IP, CDKN1C, **PTEN**, SMAD3/7, PRMT5, RECK, ITGA6, NLK, DKK3, EZH2 | proliferation; migration/invasion; autophagy | (74, 75) |
| **hsa-miR-15a-5p^#^** | Tumor suppressor | **BCL2**, CCNE1, **VEGFA**, **BMI1**, MYB, **CCND1**, CRKL, MN1, CLCN3, FOXO1, WEE1, CHEK1, YAP1, RECK | proliferation; apoptosis; migration/invasion; EMT | (76-80) |
| **hsa-miR-193b-3p** | Tumor suppressor | **CCND1**, PLAU, MCL1, KRAS, RAD51, ETS1, SMAD3, MAX, NF1, ESR1, PRAP1, MYB | proliferation; migration/invasion | (81-85) |
| **hsa-mir-30b-5p** | Oncogenic | TP53BP2, CCNE2, PDGFRB, **TP53**, **BCL2** | proliferation; migration/invasion; apoptosis | (86, 87) |
| **hsa-mir-29a-3p** | Tumor suppressor/Oncogenic | CDK6, CDC42, SPARC, DNMT3A/B, MCL1, TET1, SUV420H2, **BCL2**, **PTEN**, **VEGFA**, **CCND1**, AKT2 | EMT; proliferation; metastasis | (88-90) |

**List of references for Supplementary Table S6**

1. Wu H, Zhu S, Mo YY. Suppression of cell growth and invasion by miR-205 in breast cancer. Cell Res. 2009;19(4):439-48.

2. Wu H, Mo YY. Targeting miR-205 in breast cancer. Expert Opin Ther Targets. 2009;13(12):1439-48.

3. Wang L, Kang FB, Wang J, Yang C, He DW. Downregulation of miR-205 contributes to epithelial-mesenchymal transition and invasion in triple-negative breast cancer by targeting HMGB1-RAGE signaling pathway. Anticancer Drugs. 2019;30(3):225-32.

4. Iorio MV, Casalini P, Piovan C, Di Leva G, Merlo A, Triulzi T, et al. microRNA-205 regulates HER3 in human breast cancer. Cancer Res. 2009;69(6):2195-200.

5. Gregory PA, Bert AG, Paterson EL, Barry SC, Tsykin A, Farshid G, et al. The miR-200 family and miR-205 regulate epithelial to mesenchymal transition by targeting ZEB1 and SIP1. Nat Cell Biol. 2008;10(5):593-601.

6. Greene SB, Herschkowitz JI, Rosen JM. The ups and downs of miR-205: identifying the roles of miR-205 in mammary gland development and breast cancer. RNA Biol. 2010;7(3):300-4.

7. Greene SB, Gunaratne PH, Hammond SM, Rosen JM. A putative role for microRNA-205 in mammary epithelial cell progenitors. J Cell Sci. 2010;123(Pt 4):606-18.

8. Elgamal OA, Park JK, Gusev Y, Azevedo-Pouly AC, Jiang J, Roopra A, et al. Tumor suppressive function of mir-205 in breast cancer is linked to HMGB3 regulation. PLoS One. 2013;8(10):e76402.

9. Zhao S, Han J, Zheng L, Yang Z, Zhao L, Lv Y. MicroRNA-203 Regulates Growth and Metastasis of Breast Cancer. Cell Physiol Biochem. 2015;37(1):35-42.

10. Taube JH, Malouf GG, Lu E, Sphyris N, Vijay V, Ramachandran PP, et al. Epigenetic silencing of microRNA-203 is required for EMT and cancer stem cell properties. Sci Rep. 2013;3:2687.

11. Le LT, Cazares O, Mouw JK, Chatterjee S, Macias H, Moran A, et al. Loss of miR-203 regulates cell shape and matrix adhesion through ROBO1/Rac/FAK in response to stiffness. J Cell Biol. 2016;212(6):707-19.

12. DeCastro AJ, Dunphy KA, Hutchinson J, Balboni AL, Cherukuri P, Jerry DJ, et al. MiR203 mediates subversion of stem cell properties during mammary epithelial differentiation via repression of DeltaNP63alpha and promotes mesenchymal-to-epithelial transition. Cell Death Dis. 2013;4:e514.

13. Wellner U, Schubert J, Burk UC, Schmalhofer O, Zhu F, Sonntag A, et al. The EMT-activator ZEB1 promotes tumorigenicity by repressing stemness-inhibiting microRNAs. Nat Cell Biol. 2009;11(12):1487-95.

14. McFall T, McKnight B, Rosati R, Kim S, Huang Y, Viola-Villegas N, et al. Progesterone receptor A promotes invasiveness and metastasis of luminal breast cancer by suppressing regulation of critical microRNAs by estrogen. J Biol Chem. 2018;293(4):1163-77.

15. Liu XX, Li XJ, Zhang B, Liang YJ, Zhou CX, Cao DX, et al. MicroRNA-26b is underexpressed in human breast cancer and induces cell apoptosis by targeting SLC7A11. FEBS Lett. 2011;585(9):1363-7.

16. Li J, Kong X, Zhang J, Luo Q, Li X, Fang L. MiRNA-26b inhibits proliferation by targeting PTGS2 in breast cancer. Cancer Cell Int. 2013;13(1):7.

17. Gilam A, Shai A, Ashkenazi I, Sarid LA, Drobot A, Bickel A, et al. MicroRNA regulation of progesterone receptor in breast cancer. Oncotarget. 2017;8(16):25963-76.

18. Mandujano-Tinoco EA, Garcia-Venzor A, Munoz-Galindo L, Lizarraga-Sanchez F, Favela-Orozco A, Chavez-Gutierrez E, et al. miRNA expression profile in multicellular breast cancer spheroids. Biochim Biophys Acta Mol Cell Res. 2017;1864(10):1642-55.

19. Garofalo M, Quintavalle C, Romano G, Croce CM, Condorelli G. miR221/222 in cancer: their role in tumor progression and response to therapy. Curr Mol Med. 2012;12(1):27-33.

20. Oztemur Islakoglu Y, Noyan S, Aydos A, Gur Dedeoglu B. Meta-microRNA Biomarker Signatures to Classify Breast Cancer Subtypes. OMICS. 2018;22(11):709-16.

21. Hu X, Guo J, Zheng L, Li C, Zheng TM, Tanyi JL, et al. The heterochronic microRNA let-7 inhibits cell motility by regulating the genes in the actin cytoskeleton pathway in breast cancer. Mol Cancer Res. 2013;11(3):240-50.

22. Tang W, Zhu J, Su S, Wu W, Liu Q, Su F, et al. MiR-27 as a prognostic marker for breast cancer progression and patient survival. PLoS One. 2012;7(12):e51702.

23. Tang W, Yu F, Yao H, Cui X, Jiao Y, Lin L, et al. miR-27a regulates endothelial differentiation of breast cancer stem like cells. Oncogene. 2014;33(20):2629-38.

24. Ren YQ, Fu F, Han J. MiR-27a modulates radiosensitivity of triple-negative breast cancer (TNBC) cells by targeting CDC27. Med Sci Monit. 2015;21:1297-303.

25. Ma S, Liu M, Xu Z, Li Y, Guo H, Ge Y, et al. A double feedback loop mediated by microRNA-23a/27a/24-2 regulates M1 versus M2 macrophage polarization and thus regulates cancer progression. Oncotarget. 2016;7(12):13502-19.

26. Li X, Xu M, Ding L, Tang J. MiR-27a: A Novel Biomarker and Potential Therapeutic Target in Tumors. J Cancer. 2019;10(12):2836-48.

27. Kong LY, Xue M, Zhang QC, Su CF. In vivo and in vitro effects of microRNA-27a on proliferation, migration and invasion of breast cancer cells through targeting of SFRP1 gene via Wnt/beta-catenin signaling pathway. Oncotarget. 2017;8(9):15507-19.

28. Jiang G, Shi W, Fang H, Zhang X. miR27a promotes human breast cancer cell migration by inducing EMT in a FBXW7dependent manner. Mol Med Rep. 2018;18(6):5417-26.

29. Li X, Liu X, Xu W, Zhou P, Gao P, Jiang S, et al. c-MYC-regulated miR-23a/24-2/27a cluster promotes mammary carcinoma cell invasion and hepatic metastasis by targeting Sprouty2. J Biol Chem. 2013;288(25):18121-33.

30. Chen X, Wang YW, Zhu WJ, Li Y, Liu L, Yin G, et al. A 4-microRNA signature predicts lymph node metastasis and prognosis in breast cancer. Hum Pathol. 2018;76:122-32.

31. Liu Z, Miao T, Feng T, Jiang Z, Li M, Zhou L, et al. miR-451a Inhibited Cell Proliferation and Enhanced Tamoxifen Sensitive in Breast Cancer via Macrophage Migration Inhibitory Factor. Biomed Res Int. 2015;2015:207684.

32. Liu ZR, Song Y, Wan LH, Zhang YY, Zhou LM. Over-expression of miR-451a can enhance the sensitivity of breast cancer cells to tamoxifen by regulating 14-3-3zeta, estrogen receptor alpha, and autophagy. Life Sci. 2016;149:104-13.

33. Li H, Liu J, Chen J, Wang H, Yang L, Chen F, et al. A serum microRNA signature predicts trastuzumab benefit in HER2-positive metastatic breast cancer patients. Nat Commun. 2018;9(1):1614.

34. Nilsson S, Moller C, Jirstrom K, Lee A, Busch S, Lamb R, et al. Downregulation of miR-92a is associated with aggressive breast cancer features and increased tumour macrophage infiltration. PLoS One. 2012;7(4):e36051.

35. Lee JK, Park SR, Jung BK, Jeon YK, Lee YS, Kim MK, et al. Exosomes derived from mesenchymal stem cells suppress angiogenesis by down-regulating VEGF expression in breast cancer cells. PLoS One. 2013;8(12):e84256.

36. Lezina L, Purmessur N, Antonov AV, Ivanova T, Karpova E, Krishan K, et al. miR-16 and miR-26a target checkpoint kinases Wee1 and Chk1 in response to p53 activation by genotoxic stress. Cell Death Dis. 2013;4:e953.

37. Polytarchou C, Iliopoulos D, Struhl K. An integrated transcriptional regulatory circuit that reinforces the breast cancer stem cell state. Proc Natl Acad Sci U S A. 2012;109(36):14470-5.

38. Rivas MA, Venturutti L, Huang YW, Schillaci R, Huang TH, Elizalde PV. Downregulation of the tumor-suppressor miR-16 via progestin-mediated oncogenic signaling contributes to breast cancer development. Breast Cancer Res. 2012;14(3):R77.

39. Zhang N, Wang X, Huo Q, Li X, Wang H, Schneider P, et al. The oncogene metadherin modulates the apoptotic pathway based on the tumor necrosis factor superfamily member TRAIL (Tumor Necrosis Factor-related Apoptosis-inducing Ligand) in breast cancer. J Biol Chem. 2013;288(13):9396-407.

40. Cai X, Wang X, Cao C, Gao Y, Zhang S, Yang Z, et al. HBXIP-elevated methyltransferase METTL3 promotes the progression of breast cancer via inhibiting tumor suppressor let-7g. Cancer Lett. 2018;415:11-9.

41. Qian P, Zuo Z, Wu Z, Meng X, Li G, Wu Z, et al. Pivotal role of reduced let-7g expression in breast cancer invasion and metastasis. Cancer Res. 2011;71(20):6463-74.

42. Wang L, Li M, Zhou Y, Zhao Y. MicroRNA Let-7g Directly Targets Forkhead Box C2 (FOXC2) to Modulate Bone Metastasis in Breast Cancer. Open Med (Wars). 2017;12:157-62.

43. Camps C, Saini HK, Mole DR, Choudhry H, Reczko M, Guerra-Assuncao JA, et al. Integrated analysis of microRNA and mRNA expression and association with HIF binding reveals the complexity of microRNA expression regulation under hypoxia. Mol Cancer. 2014;13:28.

44. Khodadadi-Jamayran A, Akgol-Oksuz B, Afanasyeva Y, Heguy A, Thompson M, Ray K, et al. Prognostic role of elevated mir-24-3p in breast cancer and its association with the metastatic process. Oncotarget. 2018;9(16):12868-78.

45. Lu K, Wang J, Song Y, Zhao S, Liu H, Tang D, et al. miRNA-24-3p promotes cell proliferation and inhibits apoptosis in human breast cancer by targeting p27Kip1. Oncol Rep. 2015;34(2):995-1002.

46. Zhu D, Zhang X, Lin Y, Liang S, Song Z, Dong C. MT1JP inhibits tumorigenesis and enhances cisplatin sensitivity of breast cancer cells through competitively binding to miR-24-3p. Am J Transl Res. 2019;11(1):245-56.

47. Bai WD, Ye XM, Zhang MY, Zhu HY, Xi WJ, Huang X, et al. MiR-200c suppresses TGF-beta signaling and counteracts trastuzumab resistance and metastasis by targeting ZNF217 and ZEB1 in breast cancer. Int J Cancer. 2014;135(6):1356-68.

48. Burk U, Schubert J, Wellner U, Schmalhofer O, Vincan E, Spaderna S, et al. A reciprocal repression between ZEB1 and members of the miR-200 family promotes EMT and invasion in cancer cells. EMBO Rep. 2008;9(6):582-9.

49. Chang CJ, Chao CH, Xia W, Yang JY, Xiong Y, Li CW, et al. p53 regulates epithelial-mesenchymal transition and stem cell properties through modulating miRNAs. Nat Cell Biol. 2011;13(3):317-23.

50. Cochrane DR, Spoelstra NS, Howe EN, Nordeen SK, Richer JK. MicroRNA-200c mitigates invasiveness and restores sensitivity to microtubule-targeting chemotherapeutic agents. Mol Cancer Ther. 2009;8(5):1055-66.

51. Howe EN, Cochrane DR, Richer JK. Targets of miR-200c mediate suppression of cell motility and anoikis resistance. Breast Cancer Res. 2011;13(2):R45.

52. Jurmeister S, Baumann M, Balwierz A, Keklikoglou I, Ward A, Uhlmann S, et al. MicroRNA-200c represses migration and invasion of breast cancer cells by targeting actin-regulatory proteins FHOD1 and PPM1F. Mol Cell Biol. 2012;32(3):633-51.

53. Neves R, Scheel C, Weinhold S, Honisch E, Iwaniuk KM, Trompeter HI, et al. Role of DNA methylation in miR-200c/141 cluster silencing in invasive breast cancer cells. BMC Res Notes. 2010;3:219.

54. Perdigao-Henriques R, Petrocca F, Altschuler G, Thomas MP, Le MT, Tan SM, et al. miR-200 promotes the mesenchymal to epithelial transition by suppressing multiple members of the Zeb2 and Snail1 transcriptional repressor complexes. Oncogene. 2016;35(2):158-72.

55. Rokavec M, Wu W, Luo JL. IL6-mediated suppression of miR-200c directs constitutive activation of inflammatory signaling circuit driving transformation and tumorigenesis. Mol Cell. 2012;45(6):777-89.

56. Shimono Y, Zabala M, Cho RW, Lobo N, Dalerba P, Qian D, et al. Downregulation of miRNA-200c links breast cancer stem cells with normal stem cells. Cell. 2009;138(3):592-603.

57. Li D, Wang H, Song H, Xu H, Zhao B, Wu C, et al. The microRNAs miR-200b-3p and miR-429-5p target the LIMK1/CFL1 pathway to inhibit growth and motility of breast cancer cells. Oncotarget. 2017;8(49):85276-89.

58. Li X, Roslan S, Johnstone CN, Wright JA, Bracken CP, Anderson M, et al. MiR-200 can repress breast cancer metastasis through ZEB1-independent but moesin-dependent pathways. Oncogene. 2014;33(31):4077-88.

59. Lim YY, Wright JA, Attema JL, Gregory PA, Bert AG, Smith E, et al. Epigenetic modulation of the miR-200 family is associated with transition to a breast cancer stem-cell-like state. J Cell Sci. 2013;126(Pt 10):2256-66.

60. Sun L, Yao Y, Liu B, Lin Z, Lin L, Yang M, et al. MiR-200b and miR-15b regulate chemotherapy-induced epithelial-mesenchymal transition in human tongue cancer cells by targeting BMI1. Oncogene. 2012;31(4):432-45.

61. Yang X, Hu Q, Hu LX, Lin XR, Liu JQ, Lin X, et al. miR-200b regulates epithelial-mesenchymal transition of chemo-resistant breast cancer cells by targeting FN1. Discov Med. 2017;24(131):75-85.

62. Hu Y, Zhu Q, Tang L. MiR-99a antitumor activity in human breast cancer cells through targeting of mTOR expression. PLoS One. 2014;9(3):e92099.

63. Qin H, Liu W. MicroRNA-99a-5p suppresses breast cancer progression and cell-cycle pathway through downregulating CDC25A. J Cell Physiol. 2019;234(4):3526-37.

64. Turcatel G, Rubin N, El-Hashash A, Warburton D. MIR-99a and MIR-99b modulate TGF-beta induced epithelial to mesenchymal plasticity in normal murine mammary gland cells. PLoS One. 2012;7(1):e31032.

65. Wang X, Li Y, Qi W, Zhang N, Sun M, Huo Q, et al. MicroRNA-99a inhibits tumor aggressive phenotypes through regulating HOXA1 in breast cancer cells. Oncotarget. 2015;6(32):32737-47.

66. Xia M, Li H, Wang JJ, Zeng HJ, Wang SH. MiR-99a suppress proliferation, migration and invasion through regulating insulin-like growth factor 1 receptor in breast cancer. Eur Rev Med Pharmacol Sci. 2016;20(9):1755-63.

67. Yang Z, Han Y, Cheng K, Zhang G, Wang X. miR-99a directly targets the mTOR signalling pathway in breast cancer side population cells. Cell Prolif. 2014;47(6):587-95.

68. Bhat-Nakshatri P, Wang G, Collins NR, Thomson MJ, Geistlinger TR, Carroll JS, et al. Estradiol-regulated microRNAs control estradiol response in breast cancer cells. Nucleic Acids Res. 2009;37(14):4850-61.

69. Han M, Wang Y, Liu M, Bi X, Bao J, Zeng N, et al. MiR-21 regulates epithelial-mesenchymal transition phenotype and hypoxia-inducible factor-1alpha expression in third-sphere forming breast cancer stem cell-like cells. Cancer Sci. 2012;103(6):1058-64.

70. Lu Z, Liu M, Stribinskis V, Klinge CM, Ramos KS, Colburn NH, et al. MicroRNA-21 promotes cell transformation by targeting the programmed cell death 4 gene. Oncogene. 2008;27(31):4373-9.

71. Petrovic N. miR-21 Might be Involved in Breast Cancer Promotion and Invasion Rather than in Initial Events of Breast Cancer Development. Mol Diagn Ther. 2016;20(2):97-110.

72. Si ML, Zhu S, Wu H, Lu Z, Wu F, Mo YY. miR-21-mediated tumor growth. Oncogene. 2007;26(19):2799-803.

73. Yan LX, Wu QN, Zhang Y, Li YY, Liao DZ, Hou JH, et al. Knockdown of miR-21 in human breast cancer cell lines inhibits proliferation, in vitro migration and in vivo tumor growth. Breast Cancer Res. 2011;13(1):R2.

74. Liu F, Sang M, Meng L, Gu L, Liu S, Li J, et al. miR92b promotes autophagy and suppresses viability and invasion in breast cancer by targeting EZH2. Int J Oncol. 2018;53(4):1505-15.

75. Peng F, Zhang Y, Wang R, Zhou W, Zhao Z, Liang H, et al. Identification of differentially expressed miRNAs in individual breast cancer patient and application in personalized medicine. Oncogenesis. 2016;5:e194.

76. Luo Q, Li X, Li J, Kong X, Zhang J, Chen L, et al. MiR-15a is underexpressed and inhibits the cell cycle by targeting CCNE1 in breast cancer. Int J Oncol. 2013;43(4):1212-8.

77. Patel N, Garikapati KR, Makani VKK, Nair AD, Vangara N, Bhadra U, et al. Regulating BMI1 expression via miRNAs promote Mesenchymal to Epithelial Transition (MET) and sensitizes breast cancer cell to chemotherapeutic drug. PLoS One. 2018;13(2):e0190245.

78. Patel N, Garikapati KR, Pandita RK, Singh DK, Pandita TK, Bhadra U, et al. miR-15a/miR-16 down-regulates BMI1, impacting Ub-H2A mediated DNA repair and breast cancer cell sensitivity to doxorubicin. Sci Rep. 2017;7(1):4263.

79. Patel N, Garikapati KR, Ramaiah MJ, Polavarapu KK, Bhadra U, Bhadra MP. miR-15a/miR-16 induces mitochondrial dependent apoptosis in breast cancer cells by suppressing oncogene BMI1. Life Sci. 2016;164:60-70.

80. Yang L, Zhao W, Wei P, Zuo W, Zhu S. Tumor suppressor p53 induces miR-15a processing to inhibit neuronal apoptosis inhibitory protein (NAIP) in the apoptotic response DNA damage in breast cancer cell. Am J Transl Res. 2017;9(2):683-91.

81. Hulin JA, Tommasi S, Elliot D, Hu DG, Lewis BC, Mangoni AA. MiR-193b regulates breast cancer cell migration and vasculogenic mimicry by targeting dimethylarginine dimethylaminohydrolase 1. Sci Rep. 2017;7(1):13996.

82. Leivonen SK, Makela R, Ostling P, Kohonen P, Haapa-Paananen S, Kleivi K, et al. Protein lysate microarray analysis to identify microRNAs regulating estrogen receptor signaling in breast cancer cell lines. Oncogene. 2009;28(44):3926-36.

83. Li XF, Yan PJ, Shao ZM. Downregulation of miR-193b contributes to enhance urokinase-type plasminogen activator (uPA) expression and tumor progression and invasion in human breast cancer. Oncogene. 2009;28(44):3937-48.

84. Long J, Ji Z, Jiang K, Wang Z, Meng G. miR-193b Modulates Resistance to Doxorubicin in Human Breast Cancer Cells by Downregulating MCL-1. Biomed Res Int. 2015;2015:373574.

85. Tahiri A, Leivonen SK, Luders T, Steinfeld I, Ragle Aure M, Geisler J, et al. Deregulation of cancer-related miRNAs is a common event in both benign and malignant human breast tumors. Carcinogenesis. 2014;35(1):76-85.

86. Tormo E, Adam-Artigues A, Ballester S, Pineda B, Zazo S, Gonzalez-Alonso P, et al. The role of miR-26a and miR-30b in HER2+ breast cancer trastuzumab resistance and regulation of the CCNE2 gene. Sci Rep. 2017;7:41309.

87. Wu T, Song H, Xie D, Hua K, Hu J, Deng Y, et al. Mir-30b-5p Promotes Proliferation, Migration, and Invasion of Breast Cancer Cells via Targeting ASPP2. Biomed Res Int. 2020;2020:7907269.

88. Pei YF, Lei Y, Liu XQ. MiR-29a promotes cell proliferation and EMT in breast cancer by targeting ten eleven translocation 1. Biochim Biophys Acta. 2016;1862(11):2177-85.

89. Jiang H, Zhang G, Wu JH, Jiang CP. Diverse roles of miR-29 in cancer (review). Oncol Rep. 2014;31(4):1509-16.

90. Wu Y, Shi W, Tang T, Wang Y, Yin X, Chen Y, et al. miR-29a contributes to breast cancer cells epithelial-mesenchymal transition, migration, and invasion via down-regulating histone H4K20 trimethylation through directly targeting SUV420H2. Cell Death Dis. 2019;10(3):176.
