# Supplementary material for: The Physiological MicroRNA Landscape in Nipple Aspirate Fluid: Differences and Similarities with Breast Tissue, Breast Milk, Plasma and Serum
Source: Int J Mol Sci. 2020 Nov 11;21(22):8466. doi: 10.3390/ijms21228466 (PMC7696615; doi:10.3390/ijms21228466)
Supplement: Supplementary file 1 [file ijms-21-08466-s001.zip › Supplementary Table S7.docx]

**Supplementary Table S7. Unique physiological roles and pathway involvement to each set of specimen-specific miRNAs (Table 2) and for the 7 shared miRNAs (bold Table 1).** Data was retrieved from miRPathDB v2.0 using the KEGG, WikiPathway and GO biological processes databases. Stringent criteria for miRPathDB query included selection of data based on strong experimental evidence and with at least >50% significant miRNAs per specimen in each pathway (Supplementary Table A7A). Physiological roles and pathways that were shared with more than one specimen type were not included (Supplementary Table A7B).

| **Supplementary Table A7A** | | | | | |
| --- | --- | --- | --- | --- | --- |
| **Serum** | **Plasma** | **Breast milk** | **NAF** | **Breast tissue** | **7 shared miRNAs** |
| miRNAs in cancer | cell cycle | miRNAs in cancer | pathways in cancer | cell cyle | DNA damage response |
| angiopoietin like regulatory pathway | retinoblastoma gene in cancer | prostate cancer | bladder cancer | prostate cancer | TGF beta signaling |
| protein and metabolism regulation |  | DNA damage response | cell cycle | TGF beta signaling pathway | miRNAs involved in DNA damage response |
|  |  | **breast cancer pathway** | microRNAs in cancer | regulation of RNA metabolic process | integrated cancer pathways |
|  |  | factors and pathways | small cell lung cancer | regulation of nitrogen compound metabolic process | hepatitis C and hepatocellular carcinoma |
|  |  | senescence and autophagy | hepatitis B | regulation of gene expression | senescence and autophagy in cancer |
|  |  | DNA damage response | colorectal cancer | regulation of metabolic process | signaling pathways in glioblastoma |
|  |  | factors and pathways | chronic myeloid leukemia |  | DNA damage response |
|  |  | senescence and autophagy | HTLV infection |  | miRNA regulation of DNA damage response |
|  |  |  | prostate cancer |  | cell cycle |
|  |  |  | melanoma |  | retinoblastoma gene in cancer |
|  |  |  | pancreatic cancer |  | hepatitis B |
|  |  |  | pancreatic adenocarcinoma pathway |  | miRNAs in cancer |
|  |  |  | bladder cancer |  | cell cycle |
|  |  |  | senescence and authophagy in cancer |  | p53 signaling pathway |
|  |  |  | **breast cancer pathway** |  | bladder cancer |
|  |  |  | chromosomal and microsatellite instability in colorectal cancer |  | non-small cell lung cancer |
|  |  |  | DNA damage response (only ATM dependent) |  | HTLV infection |
|  |  |  | **integrated breast cancer pathway** |  | chronic myeloid leukemia |
|  |  |  | cell cyle |  | FoxO signaling pathway |
|  |  |  | signalling pathways in glioblastoma |  | transcription by RNA polymerase II |
|  |  |  | cell population proliferation |  | positive regulation of gene expression |
|  |  |  | positive regulation of cell population proliferation |  | regulation of nucleic acid templated transcription |
|  |  |  |  |  | transcription DNA templated |
|  |  |  |  |  | signal transduction by p53 class mediator |
|  |  |  |  |  | regulation of nitrogen compound metabolic process |
|  |  |  |  |  | positive regulation of cellular metabolic process |
|  |  |  |  |  | cellular metabolic process |
|  |  |  |  |  | cell cycle |
|  |  |  |  |  | cellular nitrogen compound metabolic process |
|  |  |  |  |  | gene expression |

| **Supplementary Table A7B** | | |
| --- | --- | --- |
| Serum | 2 | protein and metabolism regulation  angiopoietin-like regulatory pathway |
| Breast milk | 2 | senescence and autophagy  factors and pathways |
| NAF | 11 | positive regulation of cell population  cell population proliferation  proliferation DNA damage response (only ATM dependent)  pathways in cancer  **integrated breast cancer pathway**  senescence and authophagy in cancer pancreatic cancer  melanoma  pancreatic adenocarcinoma pathway  small cell lung cancer  colorectal cancer  chromosomal and microsatellite instability in colorectal cancer |
| Breast tissue | 4 | TGF beta signaling pathway  regulation of metabolic process  regulation of gene expression regulation of RNA metabolic process |
| 7 shared miRNAs | 18 | integrated cancer pathways  senescence and autophagy in cancer  p53 signaling pathway  signal transduction by p53 class mediator  miRNAs involved in DNA damage response  gene expression  transcription DNA template  FoxO signaling pathway  regulation of nucleic acid templated transcription  non-small cell lung cancer  cellular nitrogen compound  metabolic process  cellular metabolic process  transcription by RNA polymerase II  positive regulation of gene expression  miRNA regulation of DNA damage response  TGF beta signaling  positive regulation of cellular metabolic process  hepatitis C and hepatocellular carcinoma |
